# Use of Industrial Wastes as Sustainable Nutrient Sources for Bacterial Cellulose (BC) Production: Mechanism, Advances, and Future Perspectives

**DOI:** 10.3390/polym13193365

**Published:** 2021-09-30

**Authors:** Abudukeremu Kadier, R. A. Ilyas, M. R. M. Huzaifah, Nani Harihastuti, S. M. Sapuan, M. M. Harussani, M. N. M. Azlin, Rustiana Yuliasni, R. Ibrahim, M. S. N. Atikah, Junying Wang, K. Chandrasekhar, M Amirul Islam, Shubham Sharma, Sneh Punia, Aruliah Rajasekar, M. R. M. Asyraf, M. R. Ishak

**Affiliations:** 1 Laboratory of Environmental Science and Technology, The Xinjiang Technical Institute of Physics and Chemistry, Key Laboratory of Functional Materials and Devices for Special Environments, Chinese Academy of Sciences, Urumqi 830011, China; abudukeremu@ms.xjb.ac.cn (A.K.); wangjunying19@mails.ucas.ac.cn (J.W.); 2 School of Chemical and Energy Engineering, Faculty of Engineering, Universiti Teknologi Malaysia (UTM), Johor Bahru 81310, Johor, Malaysia; 3 Centre for Advanced Composite Materials (CACM), Universiti Teknologi Malaysia (UTM), Johor Bahru 81310, Johor, Malaysia; 4 Faculty of Agricultural Science and Forestry, Bintulu Campus, Universiti Putra Malaysia, Bintulu 97000, Sarawak, Malaysia; 5 Centre of Industrial Pollution Prevention Technology, The Ministry of Industry, Jawa Tengah 50136, Indonesia; nanisoeharto@yahoo.com (N.H.); rustianay@yahoo.com (R.Y.); 6 Advanced Engineering Materials and Composites Research Centre (AEMC), Department of Mechanical and Manufacturing Engineering, Faculty of Engineering, Universiti Putra Malaysia (UPM), Serdang 43400, Selangor, Malaysia; sapuan@upm.edu.my (S.M.S.); mmharussani17@gmail.com (M.M.H.); 7 Laboratory of Technology Biocomposite, Institute of Tropical Forestry and Forest Products (INTROP), Universiti Putra Malaysia (UPM), Serdang 43400, Selangor, Malaysia; mohdazlin@uitm.edu.my; 8 Department of Textile Technology, School of Industrial Technology, Universiti Teknologi MARA, Universiti Teknologi Mara Negeri Sembilan, Kuala Pilah 72000, Negeri Sembilan, Malaysia; 9 Innovation & Commercialization Division, Forest Research Institute Malaysia, Kepong 52109, Selangor Darul Ehsan, Malaysia; rushdan@frim.gov.my; 10 Department of Chemical and Environmental Engineering Engineering, Faculty of Engineering, Universiti Putra Malaysia (UPM), Serdang 43400, Selangor, Malaysia; sitinuratikah_asper7@yahoo.com; 11 School of Civil and Environmental Engineering, Yonsei University, Seoul 03722, Korea; chanduibt@gmail.com; 12 Laboratory for Quantum Semiconductors and Photon-Based BioNanotechnology, Department of Electrical and Computer Engineering, Faculty of Engineering, Université de Sherbrooke, Sherbrooke, QC J1K 2R1, Canada; amirul.geb@gmail.com; 13 Department of Mechanical Engineering, IK Gujral Punjab Technical University, Jalandhar 144001, India; shubham543sharma@gmail.com; 14 Department of Food, Nutrition and Packaging Sciences, Clemson University, Clemson, SC 29634, USA; snehpunia69@gmail.com; 15 Environmental Molecular Microbiology Research Laboratory, Department of Biotechnology, Thiruvalluvar University, Serkkadu, Vellore 632115, India; 16 Department of Aerospace Engineering, Universiti Putra Malaysia (UPM), Serdang 43400, Selangor, Malaysia; asyrafriz96@gmail.com (M.R.M.A.); mohdridzwan@upm.edu.my (M.R.I.)

**Keywords:** bacterial cellulose (BC), biopolymer, industrial waste, microbial cellulose, carbon source, nitrogen source

## Abstract

A novel nanomaterial, bacterial cellulose (BC), has become noteworthy recently due to its better physicochemical properties and biodegradability, which are desirable for various applications. Since cost is a significant limitation in the production of cellulose, current efforts are focused on the use of industrial waste as a cost-effective substrate for the synthesis of BC or microbial cellulose. The utilization of industrial wastes and byproduct streams as fermentation media could improve the cost-competitiveness of BC production. This paper examines the feasibility of using typical wastes generated by industry sectors as sources of nutrients (carbon and nitrogen) for the commercial-scale production of BC. Numerous preliminary findings in the literature data have revealed the potential to yield a high concentration of BC from various industrial wastes. These findings indicated the need to optimize culture conditions, aiming for improved large-scale production of BC from waste streams.

## 1. Introduction

As a novel nanomaterial, Bacterial Cellulose (BC) has continued to draw scholarly interests since it was first discovered due to its unique properties, such as a high degree of purity, biodegradability, biocompatibility, and ease of polymerisation [1,2], making BC a material with a wide range of applications including skin and bone tissue engineering, barrier technology, and electrical, electrochemical, and sensing applications [3,4,5,6,7,8]. Despite offering many beneficial properties, its expensive production cost bounds its industrial-scale application. Conventionally, producers utilize fructose and glycerol as conventional carbon sources, however, the costs of these materials are remarkably high. A growing research body studies methods of minimizing the BC production cost. However, it has ended up with unconvincing and inadequate findings [9]. Recent research on reducing the production costs has emphasized utilizing waste products for sources of carbon or nitrogen. At present, active research to investigate the cost-effectiveness of BC synthesis from different waste products is ongoing and needs to be elaborated.

Nevertheless, the literature analysis compiles crucial developments in the field and, hence, enables the assessment of the future practicability of this manufacturing of BC for various applications [10,11,12]. The feasibility of using waste in BC production is examined in this paper through an extensive literature review to strengthen the current phase of knowledge and analyse discernible trends and gaps in inexperience. Many industrial wastes are rich in carbon and nitrogen content; hence, utilizing them as substrates may yield high microbial cellulose concentrations with the optimization of culture conditions.

## 2. Overview of Bacterial Cellulose (BC) and Its Applications

**Figure 1:**
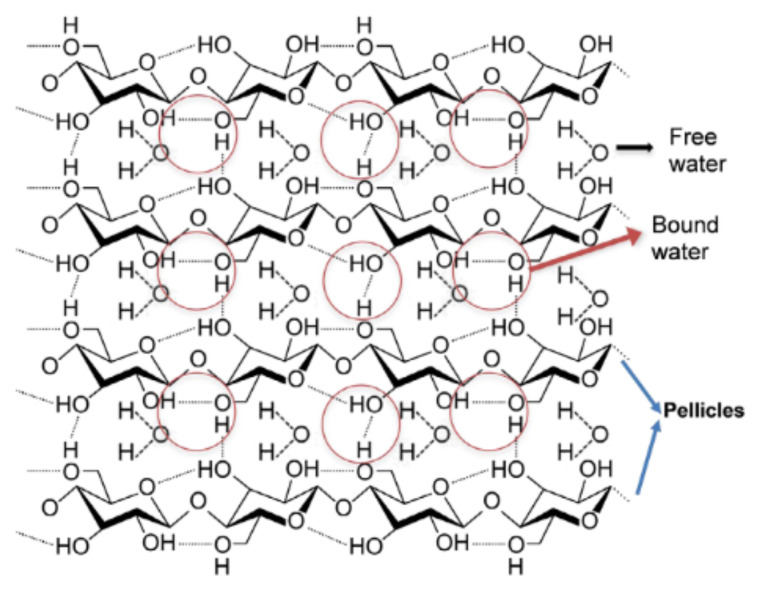
Schematic of the molecular structure of bacterial cellulose and its bound and free water [23].

Bacterial cellulose (BC), commonly known as biocellulose, which is the purest form of cellulose, continues to receive widespread focus due to its superior physicochemical properties compared to plant cellulose, in which impurities such as hemicellulose and lignin are often found [13,14,15,16,17,18,19,20,21]. Some of the superior physicochemical properties of BC include high tensile strength, crystallinity, and water holding capacity (WHC), as well as a slow water release rate (WRR) and remarkable moldability into three-dimensional structures [22]. The water molecules are bonded through hydrogen bonds within the complex structure of BC. The unbonded free water molecules will penetrate and exit the BC molecular structure, as shown in Figure 1 [23].

**Figure 2:**
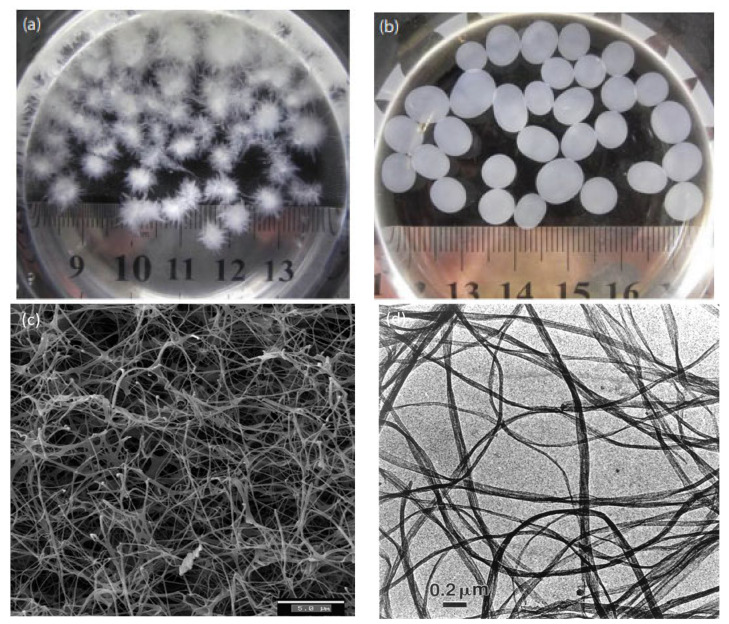
Optical images (**a**,**b**), scanning electron microscope (SEM) images (**c**) of BC samples and ultrastructural transmission electron microscopy (TEM) images (**d**) of BC samples [24,27,28].

**Figure 3:**
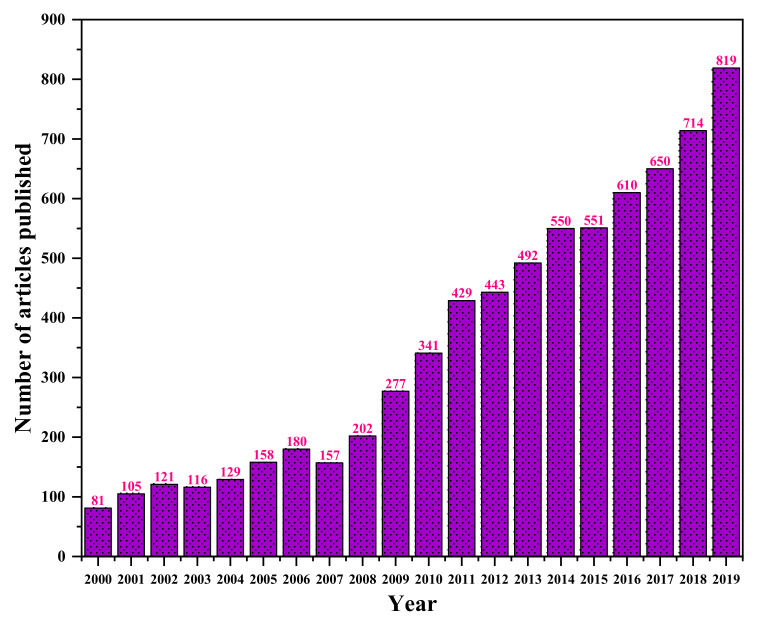
Number of publications on bacterial cellulose since 2000–2020 (Scopus search engine system, the search term “bacterial cellulose”).

Bi et al. [24] characterized the BC synthesized from different strains in agitated culture. The macrostructure morphology of BC varied depending on the different culture methods [25]. The research used isolated bacteria, namely *Komagataeibacter nataicola Y19* (BC-1) and *Gluconacetobacter entanii* ACCC10215 (BC-2). Both bacteria were fermented in Hestrin–Schramm medium. The BC morphology result depicts that both samples have different sizes and shapes, as shown in Figure 2. Figure 2a,b illustrates the optical image of BC-1 and BC2, while Figure 2c,d shows the morphology of BC samples. The BC-1 shows the flocky asterisk-like and solid sphere-like for BC-2. In addition, based on Pang et al. [7], BC is useful as a natural renewable polymer in many fields due to its versatility and numerous notable properties such as biocompatibility, chirality, structure-forming potential, hydrophilicity, high crystallinity, high purity, a high degree of polymerization, high porosity, large specific area, favourable permeability, flexibility, hygroscopicity, and biodegradability. BC is produced as extrusions of glucose chains from the bacterial body via small pores present on their cell envelope. These extrusions then form microfibrils that further aggregate into web-shaped cellulose ribbon networks with many empty spaces between the fibres. The well-separated non-fibrils of BC create an expanded surface area and highly porous matrix. The basic fibril structure contains a β-1→4 glucan chain with the molecular formula (C_6_H_10_O_5_)_n_ and is held together by hydrogen bonds. These microfibrils are approximately 100-fold smaller than the fibrils of vegetal cellulose [26]. Until recently, much research was done on the production of BC and its modification and applications in various fields. As displayed in Figure 3, the number of BC publications has increased since 2000 from 81 to 819 publications.

**Figure 4:**
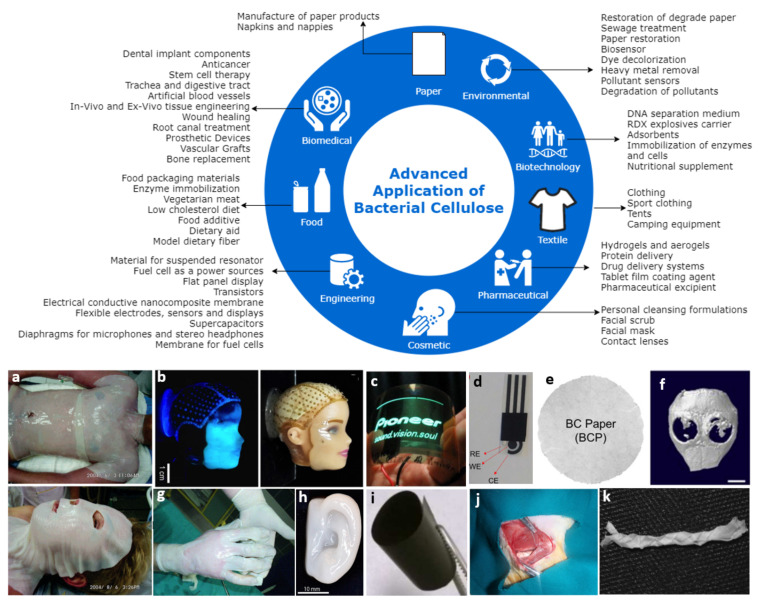
Advanced application of bacterial cellulose (BC). (**a**) A never-dried microbial cellulose membrane shows remarkable conformability to the various body contours, maintains a moist environment, and significantly reduces pain [74]. (**b**) A doll face was scanned, and a 4.5 wt % Flink containing A. xylinum was deposited onto the face using a custom-built 3D printer. In situ cellulose growth leads to the formation of a cellulose-reinforced hydrogel that, after removal of all biological residues, can serve as a skin transplant [75]. (**c**) Luminescence of an organic light-emitting diode deposited onto a flexible, low-CTE, and optically transparent cellulose nanocomposite [76]. (**d**) Screen-printed electrodes made on BC substrate [77]. (**e**) BC paper [78]. (**f**) Bone regeneration efficacy of the scaffolds [79]. (**g**) Microbial cellulose dressing applied on a wounded hand [80]. (**h**) 3D Bioprinting Human Chondrocytes with nanocellulose−alginate bioink [81]. (**i**) Flexible freestanding nanocellulose paper-based Si Anodes for Lithium-ion batteries [82]. (**j**) Cellulose acetate/poly lactic acid coaxial wet-electrospun scaffold containing citalopram-loaded gelatin nanocarriers for neural tissue [83]. (**k**) Artificial Bacterial cellulose ligament or tendons [84].

The unique macro-physical and outstanding thermal and mechanical properties of BC make it an ideal material to be applied in various fields of applications (Figure 4). BC possesses good thermal stability and low or no chronic inflammatory response, which has attracted huge attention for BC as a novel functional material in applications such as nonwoven fabric-like products and paper [29]. BC is also used as a binder in advanced paper technology due to its nano-sized structure, a property that significantly improves the durability and strength of pulp when reinforced into paper [30]. One of the main reasons it is being used in biomedicine is its excellent biocompatibility [14]. In addition, the weight-average degree of polymerization (DPw) of BC is high, such as the DPw of BC produce by *Acetobacter xylinum* BPR2001, which remained in the range of 14,000 of 16,000 [7,31]. BC possesses nanofibrillar and ultrafine structured material with an excellent combination of properties such as high flexibility and tensile strength (Young modulus of 114 GPa) [32], as well as high crystallinity (84–89%) [33]. Therefore, due to its outstanding mechanical properties, BC nanocomposites had been fabricated by reinforcing it with other polymers to be developed in various applications, including paper [29], treating tympanic membrane perforation [34,35], shielding film [36], food packaging films [37], audio speaker diaphragms [38], and so on. Development of BC for paper products had been actively conducted by Ajinomoto Corporation along with Mitsubishi Paper Mills in Japan since 1995 (JP patent 63295793) [39].

**Figure 5:**
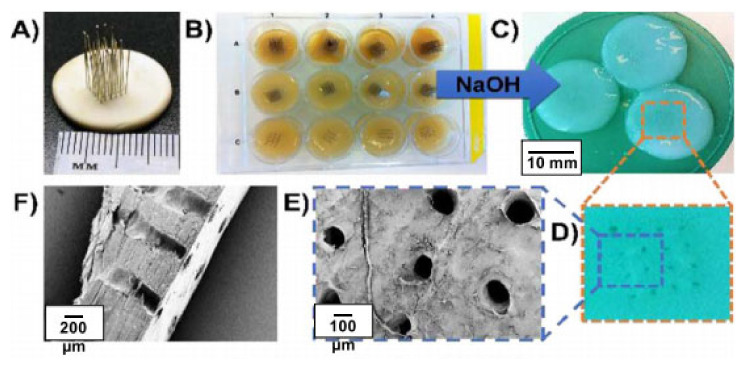
Experimental images of (**A**) clay-needle template with needles at the centre, (**B**) growing BC scaffolds with aid from clay-needle templates in static cultures, and (**C**) clean BC pellicles with channels to be an effective hydrogel-like material for different tissue engineering applications. (**D**) Enlargement of the channel area in (**C**). The channel diameter was approximately 250 μm and the inter-distance approximately 1 mm. (**E**) Scanning electron images (SEM) of channeled area in (**C**). (**F**) Cross-section of channels [85].

Due to the high porosity combined with a large specific area of three-dimensional structure, research on BC has opened up opportunities for it to be used as a photocatalyst [40], electronic sensing platform [41], and biosensing material [42,43] (Figure 5). BC has also been used widely in biomedical applications such as wound-dressing [44,45,46,47] (applied on the wounded torso, hand, and face) and cell culture [48,49,50,51] because of its excellent flexibility, high mechanical strength at wet state, water holding capacity, very low risk for irritation due to its ultra-high purity, hygroscopicity, liquid/gasses permeability, and ease of wound inspection due to its transparency. Biopolymer such as polylactic acid (PLA), starch, polyhydroxyalkanoate (PHA) [52,53,54,55,56], and synthetic polymer such as polyvinyl alcohol (PVA) and unsaturated polyester (UP) [57,58] are potential polymers to be reinforced with BC. The outstanding properties of BC such as biodegradability, good controllability during BC production, and possessing net-like morphology that is almost similar to human collagen as a biomimetic feature makes it favoured in the medical field and has been widely utilized in controlled drug delivery [59], medical pads [41], artificial skin [7,60], cartilage [61] and bone [62,63], bone tissue engineering scaffolds [64,65,66], hormones [72], and nerve guides for spinal cord injury treatment [73]. vascular grafting [67,68], blood vessel tubes [69,70], dental implant [71], proteins and hormones [72], and nerve guides for spinal cord injury treatment [73].

BC possess large surface areas and have the capability to absorb liquids. Hence, a small amount of BC can be utilized for producing coating, thickening, and binding agents, especially in the food industry. Remarkably, in 1992, BC was categorized as ‘‘generally recognized as safe’’ (GRAS) by the USA Food and Drug Administration (FDA) and, hence, is suitable to be used in food industry applications [86]. The largest industrial-scale production of BC that has been produced so far is led by Cetus Co. (Emeryville, CA, USA) and Weyerhaeuser Co. (Tacoma, Washington, DC, USA). Both companies develop a Cellulon, a bulking agent with a wide range of applications such as in coating, binding, and thickening applications [87]. Besides that, BC also can be used in the oil and gas recovery sector, cosmetics, adhesives, paints, and mining. High-end audio speaker systems had been fabricated by Sony Corporation using BC. This might be due to its good acoustic properties [88].

**Figure 6:**
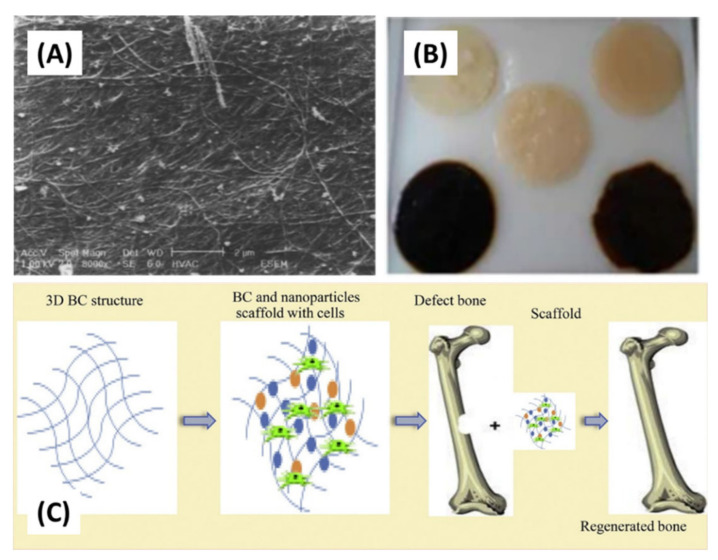
(**A**) Network structure of ribbon-shaped fibrils of BC, (**B**) natural biomaterial of BC, and (**C**) 3D-shaped BC for bone tissue engineering [87,89].

Food packing, battery separator, transparent coating or film, pharmaceutical industries, adsorbent, cosmetics, water treatment, ethanol production, biomaterials, artificial blood vessels, electric conductors or magnetic materials, and scaffolds for tissue engineering are examples of uses of BC in industrial and medical areas [10,90]. This can be observed in Figure 6. Besides that, BC has been utilized in biomedical applications such as scaffolds and ex-situ and in situ modified through different processes [91]. The culture conditions are modified with additives or reinforcement materials via in situ modification, whereas the modification of ex-situ is performed after BC harvest. Incorporation of the additive materials can be added into a growing BC microfibril for the preparation of BC composites with required properties. A biocomposite is a material composed of two or more distinct constituent materials (one being naturally derived) which are combined to yield a new material with improved performance over single constituent materials [92,93,94,95]. This modification type can be employed in a static method for the purpose of control of properties, shape, and structure of modified BC. This application is mostly applied in bone tissue engineering, in which, in order to produce BC scaffolds with microporous structure, paraffin wax microspheres were added into culture medium via an in situ modification technique [96].

**Figure 7:**
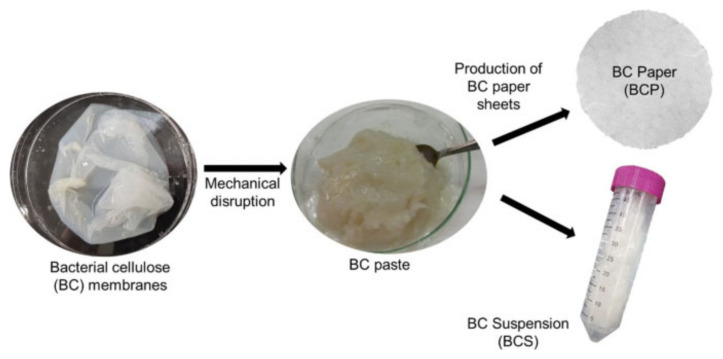
Schematic diagram to explain the approach for bacterial cellulose matrices production [78].

Gonçalves-Pimentel et al. [97] conducted experiments on BC as a support for the growth of retinal pigment epithelium, showing that all surface-modified BC substrates showed similar permeation coefficients with solutes of up to 300 kDa. Surface modification of BC greatly improved the proliferation and adhesion of retinal pigment epithelium cells. All samples showed. Insignificant stress−strain behaviour was observed in all samples, of which acetylated BC showed the highest elastic modulus; however, after some period, it exhibited a slightly smaller tensile strength and elongation at break as compared to control BC [98]. A study conducted by Buruaga-Ramiro [78] on the suitability of BC matrices to prepare enzymatically active nanocomposites shows improvement in durability, reusability, and thermal stability of BC/lipase nanocomposites (Figure 7). Besides that, the enzyme immobilised onto BC/lipase nanocomposites paper retained 60% of its activity after 48 h at 60 °C. The results attained suggest that BC/lipase nanocomposites are promising biomaterials for the development of green biotechnological devices with potential applications to be used as part of biosensor devices with applications in many fields such as food quality control, environmental monitoring, and clinical diagnosis.

The effect of BC on disintegrability in composting conditions of plasticized polyhydroxybutyrate (PHB) nanocomposites [99] shows that the compounds with BC and plasticizer presented a similar behaviour to that of control plasticized PHB. This might be due to the low dispersion and low interfacial adhesion of BC in the matrix. However, the crystallinity of PHB nanocomposites was increased. Another study conducted by Zhang et al. [100] on the reinforcement of BC with polyvinyl alcohol (PVA) coated with Bichar-Nanosilver (C-Ag) antibacterial composite membranes, shows that the BC was homogeneously mixed into the PVA gel and that the C-Ag particles were uniformly immobilized in the PVA/BC hybrid composites membrane. These hybrid composites show excellent antibacterial activity against *Escherichia coli* and good reusability to be used as drinking water treatment applications. Hamedi et al. [101] conducted experiments on double-network antibacterial hydrogel based on aminated BC and schizophyllan (SPG) biopolymer nanocomposites. A novel hydrogel composed of BC/SPG biopolymers shows an improvement in antibacterial, swelling, and mechanical properties. MTT assay displayed that amine-grafted BC/SPG stimulated the proliferation of normal human fibroblast cells. They concluded that this novel nanocomposite can be utilized in diverse areas such as anti-wrinkle dressing masks, wound healing, and absorption biomaterial for water treatment applications.

A hybrid of BCNCs–AgNPs/alginate–MoO_3_NPs was effectively developed for H_2_S gas sensors [102]. In this study, BC was produced by *Gluconacetobacter xylinus* strain under static culture. The bionanocomposites film was successfully fabricated using a solution casting method and has the ability to detect H_2_S gas emission. Through the shift in the oxidation number of MoO_3_NPs, the colour of the film was changed. Once activated by AgNPs, MoO_3_NPs were readily reduced to a coloured sub-oxide by atomic hydrogen that was produced and received from the reaction of H_2_S gas [102].

Cazón et al. [3] conducted a study on BC reinforced polyvinyl alcohol (PVOH) composite film with eco-friendly UV-protective properties. The addition of PVOH shows improvement in mechanical and transparency properties and reduced the water vapour permeability of composite films. Thus, they concluded that these novel composite films have huge advantages to be utilized in the food industry to prevent oxidation of proteins, lipids, and vitamins, as well as the degradation of antioxidants in foods. Besides that, it can be s substitute novel material to antioxidants to increase food shelf-life as well as to maintain the quality of food products [3]. AgNP produced using CUR:HPβCD (cAgNP) reinforced with BC-based hydrogels for wound dressing applications has been developed by Gupta et al. [103]. The composites show high cytocompatibility between cAgNp and BC with high moisture content and a good level of transparency. These hydrogels-based composites also showed broad-spectrum antimicrobial activity along with antioxidant properties.

In terms of electrical applications, there are a few applications of BC, as bioelectrical devices are hard to fabricate. However, several previous works have been conducted. According to Di Pasquale et al. [104], electrodes of the sensor are made of BC that has been treated with ionic solutions and coated with conducting polymers. The mechano-electric transduction properties of the composite are used to create a generating sensor. The device, which is placed in a cantilever arrangement, is used to detect anchor acceleration. On the other hand, Di Pasquale and co-researchers are testing an all-organic Bacterial Cellulose-Conducting Polymer (BC)-PEDOT:PSS composite soaked with Ionic Liquids (ILs) as a mass sensor [104]. As a result of the applied deformation, the sensor functions as a vibrating transducer in a cantilever arrangement, producing a voltage signal. The effect of the additional mass on the system’s frequency response is used to estimate the value of the measurand. The sensing system is made of low-cost, flexible, and environmentally friendly components that may be used to create smart ubiquitous sensing systems in the future.

Wang et al. [105] created a novel wound care system that uses an aligned bacterial cellulose (BC)/gelatin membrane in combination with EF stimulation to direct cell migration and improve wound healing. The produced BC/gelatin membranes had a well-aligned fibre structure, a strong mechanical property, a high thermal stability, good light transmittance, foldability, and surface roughness, and great biocompatibility. Especially, the 40% stretched BC/gelatin membrane promoted the adhesion, orientation, and migration of NIH3T3 cells in vitro. For further increase in electrical conductivity and cell survival of polyaniline (PANI) coated BC nanocomposites, BC fibres are chemically functionalized with a poly(4-vinylaniline) (PVAN) interlayer [106]. PVAN was discovered to have increased PANI yield by promoting the creation of a uniform PANI layer with nanofiber- and nanorod-like supramolecular structures. These new electrically conductive BC/PVAN/PANI nanocomposites have the potential to enable a wide range of biomedical applications, including bioelectronic interfaces and the manufacturing of biosensors. Table 1 displays BC and its biocomposites yielded in static and agitation/shaking culture bioreactor and their various applications.

## 3. Principal Pathways of Cellulose Production

**Figure 8:**
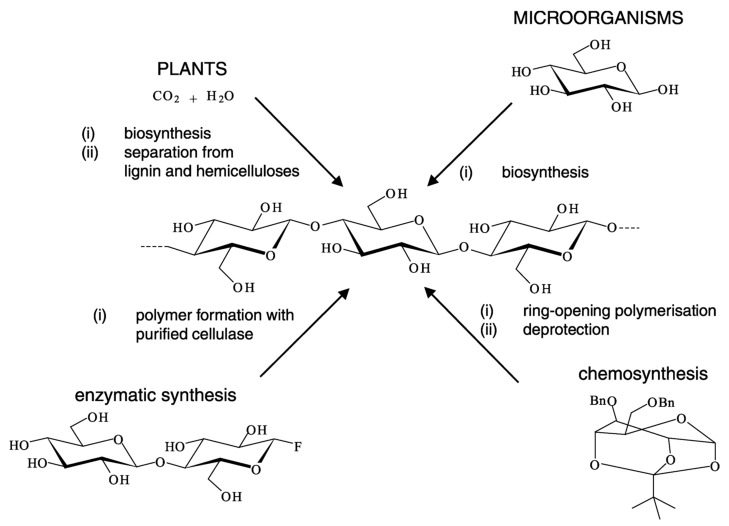
Major pathways to the cellulose [112].

Biopolymer cellulose can be produced using four distinguishing methods, including cellulose extraction, cellulose biosynthesis, enzymatic synthesis, and chemosynthesis. The most well-known method is cellulose extraction from plants, including the elimination of lignin and hemicelluloses using alkali or acid treatments. According to Klemm et al. [112], there are two main sources in cellulose production including plants and microorganisms, as shown in Figure 1. Extensive research has been conducted on the extraction of cellulose fibre from various plant fibre, i.e., sugar palm fibre [130,131,132,133,134,135,136,137,138], water hyacinth [139], ginger fibre [140,141], kenaf [142], sugarcane [143,144], lemongrass [145], cassava, corn, oat, palm oil fibre, and others [146,147,148]. Next is cellulose biosynthesis by using different types of microorganisms; (i) ***bacteria*** (gram-negative: *Alcaligenes* [149], *Salmonella*, *Enterobacter*, *Pseudomonas* [150], *Gluconacetobacter xylinus* [151], *Agrobacterium* [152], *Komagataeibacter Medellinensis* [153], *Aerobacter*, *Achromobacter insuavis* [154], *Rhizobium leguminosarum* [155], *Acetobacter* spp. [156], *Acetobacter xylinum* [157], *Zoogloea* [97], and gram-positive: *Sarcina ventriculi* [158], *Leifsonia sp* [159], *Rhizosphere bacterium*, *Bacillus subtilis* [70,160]); (ii) ***fungi*** (*Aspergillus ornatus* [161], *Penicillium* sp. [162,163], *Aspergillus terreus* MS105 [164], *Aspergillus terreus* M1 [165], *Aspergillus niger*, *Rhizopus* sp. [166,167], *Aspergillus niger* [168], *Trichoderma longibrachiatum* [169], *Beauveria Bassiana* [170], *Ascomycota* [171,172], or *Basidiomycota* [173]); (iii) ***algae*** (*Gelidium elegans* [174], *Posidonia oceanica* [175], *Aegagropila Linnaei* [176], *Komagataeibacter hansenii* [177], *Cladophora glomerata* [178]). However, extracellular synthesized cellulose as fibres is not achievable in some microorganisms. From the scientific viewpoint, the first enzymatic in vitro synthesis was initiated from cellobiosyl fluoride [179,180], and the earliest chemosynthesis was started from glucose via ring-opening polymerization of benzylated and pivaloylated derivatives [181]. These principle paths are schematically described in Figure 8 [112].

Different modified methods or additives have been applied to enhance BC production. BC gained from bioreactors have been characterized and analysed for structure, shape, and properties of BC, thermogravimetric analysis, density, porosity, yield, water holding capacity, Fourier transform infrared, purity, zeta potential, degree of polymerization, surface area, chemical structure, pore size and distribution, degree of crystallinity, and microstructure, as well as macroscopic morphology [86,182].

## 4. Fundamentals of Bacterial Cellulose (BC) Production Process

**Figure 9:**
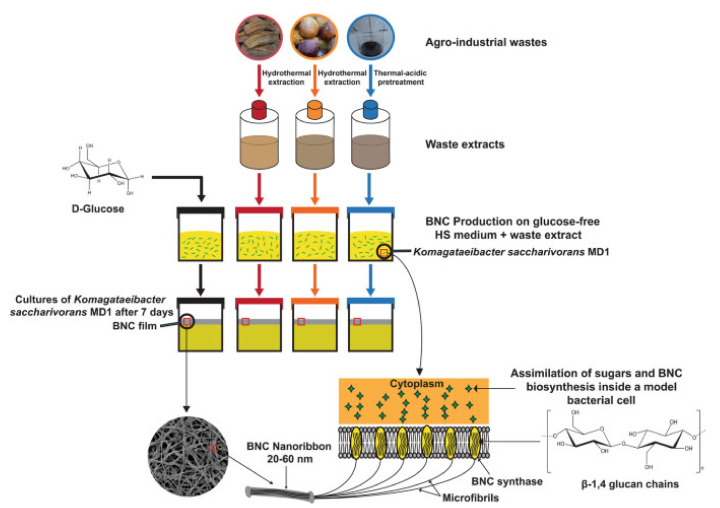
Schematic illustrations of pre-treatment of wastes for BC biosynthesis [184].

Numerous aerobic and non-pathogenic bacteria yield BC from the genera *Gluconacetobacter*, *Sarcina*, *Rhizobium*, and *Agrobacterium* either in synthetic or non-synthetic media [22]. However, these bacteria are non-photosynthetic; therefore, they need glucose or organic substrate synthesized by the photosynthetic organism to accumulate their cellulose [183]. BC production comprises fermentation in static or agitated conditions. Among the cultivation media, the most frequently used cultivation medium is a chemically defined medium known as the Hestrin–Schramm (HS) medium [22]. This medium involves somewhat expensive additional components, such as peptone, yeast extract, citric acid, glucose, and disodium phosphate, resulting in costly production. According to Abol-Fotouh et al. [184], thermal-acidic pre-treatment was proposed to enhance the characteristics of molasses, boost its (glucose-fructose) content per volume, and remove the majority of contaminants that might stifle microbial development or reduce product output, as shown in Figure 9 [185,186]. The function of thermal acidic pre-treatment of molasses in virtually complete breakdown of the contained sucrose to its original constituents, glucose and fructose, was clarified by Bae and Shoda [187].

**Figure 10:**
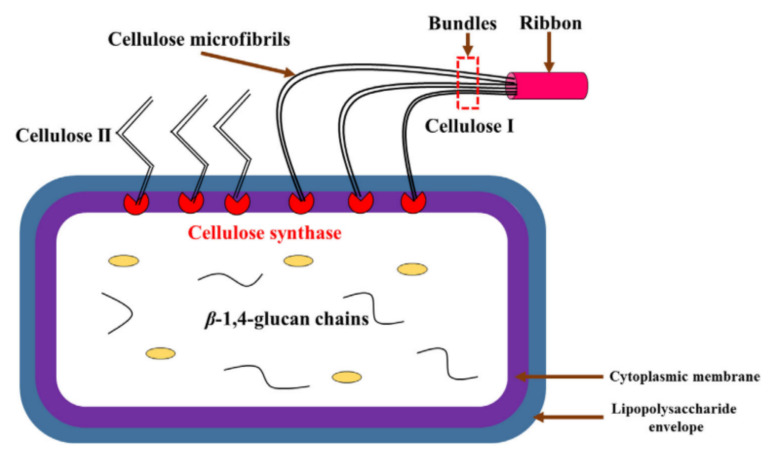
Representation of cellulose chains formation in microbial cells, and formation of micro- and macro fibrils, bundles, and ribbons [188].

Alteration of growth conditions; temperature, pH, and sources of carbon and their concentrations influenced both the quality and quantity of BC yielded. In addition, different cultivation pathways led to the production of BC with different properties and structures [22]. Figure 10 illustrates the mechanism of bacterial cellulose synthesis from *G. oxydans* on the surface cell of cellulose [188].

The BC obtained after the fermentation process has yielded good properties for several applications. Stable and efficient bacteria strains will influence the effectiveness of bacterial cellulose (BC) production. The hydroxyl groups in the BC structure have enabled direct modification by introducing other polymers into the BC network [189]. However, some modifications can be done on BC by combining other materials into the polymeric system for a broader range of applications [188]. The modification process can be divided into two main groups, which are in situ and ex-situ modifications. An inadequate supply of oxygen causes bacteria to be inactive, which is a significant constraint in static production environments. Agitated conditions result in higher yields; however, the BC formation mechanism remains uncertain under different conditions [190].

More comprehensive applications of BC depend on practical considerations regarding production costs and scale-up capability. Recently, many studies have focused on cheap nutrient sources, diverse strains of cellulose-producing microorganisms, and supplementary components to produce value-effective BC [26]. Many waste products from different fields, such as whey, industrial waste, wastewater, and agro-industrial waste, have been examined as alternative substrates for the enhanced production of BC. Various additives or modified methods have been used to improve the production of BC. The BC harvested from other bioreactors has been characterized in terms of structure and properties such as macroscopic morphology, microstructure, degree of crystallinity, chemical structure, polymerization degree, purity, water holding capacity, porosity, and thermogravimetric ability [191]. Table 2 shows the BC production specifications, modifications, and advantages of different reactors.

## 5. Industrial Waste Streams as Feedstock for the Production of Bacterial Cellulose

**Figure 11:**
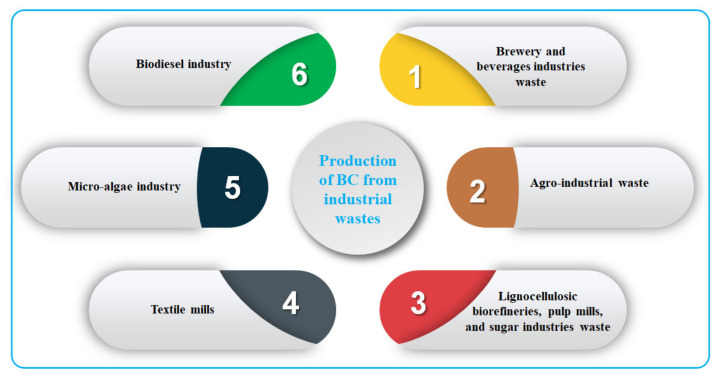
Schematic overview of Bacterial Cellulose (BC) production from different industrial wastes.

Industrial-scale applications of BC manufacturing encountered some drawbacks such as high culture medium-cost as well as low yield production. In the fermentation process, the cost of the medium for the cultivation of BC accounts for 50–65% of the total production. Thus, according to Velásquez-Riaño et al. [208] and Vazquez et al. [190], the establishment of a cost-effective culture medium for optimum product yield is important in order to enhance the process of fermentation. Some hard work has been done to minimize the production cost of BC, such as using the low-cost medium for BC cultivation and accessible and renewable sources of the nutrient. Over the last twenty years, significant global energy, environmental, and economic concerns have set the prominence of accessible and sustainable utilization of various industrial wastes such as agro-industrial waste products, wastewater treatment plants of dairy industries, brewery and beverages industries waste, waste from textile mills, waste from the micro-algae industry, etc. The innovations in clean and green technology techniques, as well as biotechnological methods, have equipped scientists and researchers with platforms for renewable natural sources consumption, i.e., using industrial waste to produce BC. The utilization of these industrial wastes for BC production helps prevent disposal and environmental pollution, aid in waste management, and, hence, reduce industrial waste disposal costs. From this novel approach, the production of BC from the industrial wastes can be categorized into six individual industrial wastes as illustrated in Figure 11: (1) brewery and beverages industries wastes; (2) agro-industrial wastes; (3) lignocellulosic biorefineries, pulp mills, and sugar industries wastes; (4) textile mills; (5) micro-algae industry wastes; (6) biodiesel industry wastes.

The examples are a small number of possible limitations of more lignocellulosic, sugar, brewery, and other industrial wastes as media without any additional nutrient source or as nitrogen and carbon sources with an additional nutrient source for the production of BC. Among all industrial waste, agro-industrial wastes are seen as highly potent and can be extensively utilized for producing BC. This might be due to the higher BC productivity and large-scale accessibility. Besides that, municipal waste is becoming a progressively more prominent source of biomass waste with high organic content, especially carbon, as a result of fast urbanization around the world, especially in developing countries [209,210]. The potential of upscale production of BC at a large scale or industrial scale from all the low-cost industries waste media are elaborated here; specifically, those that do not require complicated or complex supplementation, detoxification, and pre-treatments. Currently, the production of BC from industrial waste media has been observed to have comparable yield, physical, physico-chemical, crystallinity, and mechanical properties compared to standard media.

Industrial waste is a rich source of carbon for the bacterial synthesis of cellulose. In the past few decades, the urge to achieve ‘zero waste’ in the industrial sector has led many researchers to utilize industrial waste and byproducts as potential nutrient sources for microbial cultivation. Of many such industries, wastes and byproducts from the food, agriculture, and brewery industries are the most commonly utilised and can be a rich source of carbon [86]. All confectionery products are made of varying amounts of sugar and sugar substitutes. Since waste from confectionaries is rich in carbohydrates, this suggests that it can yield substantial amounts of carbon [211].

In terms of efficacy of BC produced by different utilized wastes, numerous researchers have extensively studied this issue. Kongruang [212] mentioned that agro-industries waste is richer in proteins, carbohydrates, and trace elements. Thus, it resulted in a higher BC productivity. Furthermore, Goelzer et al. [213] had stated that brewery and beverage industries waste mainly affects BC production, and similar influence can be seen from other different wastes such as wastewater sugar industries [214,215], lignocellulosic biorefineries wastes [216,217,218], and micro-algae biomass industries waste [177]. The results showed that utilizing pre-treated orange peel medium produced seven times more BC than using standard (HS) medium. However, structural research revealed that BC made from various wastes had a thicker and denser pack of nanofibrils, but FTIR spectra revealed no significant differences [214]. Plus, according to Fan et al. [216], in comparison to BC produced from HS medium, BC created from waste medium had no significant variations in microstructure, features, FTIR peaks, crystallinity index, or color parameter. These observations are in line with results discovered by Qi et al. [218], who found that the BC samples obtained from these hydrolysates had similar physico-chemical structural characteristics (microscopic morphology, functional groups, and crystallinity), but had a high water holding capacity and low mechanical strength. Furthermore, the physico-chemical characteristics of BC generated in various media were similar. However, when compared to HS medium, the viscosity of BC formed from molasses medium is low [217]. As a result, it may be inferred that agricultural wastes from all over the world can be used as a low-cost, readily available, and abundant feedstock for BC production.

Furthermore, fiber/textile industry waste derived hydrolysate was used as growth medium for BC production and the results showed 83% higher yield (10.8 g/L) and 79% higher tensile strength (0.070 MPa) of BC as compared to the production by glucose-based HS medium [215]. Cotton-based textile wastes were treated with the ionic liquid 1-allyl-3-methylimidazolium chloride before being hydrolyzed with enzymes. This resulted in a decreasing sugar concentration of 17 g/L in the hydrolysate. Because the natural sources used in the fiber/textile sector are often high in cellulose content, the wastes generated can be used to produce a variety of value-added products such as BC after detoxification and hydrolysis treatments [219,220].

Research by Costa et al. [26] revealed that industrial debris waste, namely sugarcane molasses, corn steep liquor (CSL), and jeans laundry effluent are rich sources of carbon and nitrogen that maintain BC production using *Gluconacetobacter hansenii* [26]. The wastes yielded a high concentration of microbial cellulose; however, the main limitation of the study was a substantial deformation observed in the product. According to Gao et al. [221], the cracks in the polymer might be due to the existence of crazing at the tip of the crack during tensile testing. Besides, BC microfibrils and nanofibrils sustained the cracks until rupture occurred [222]. The further analysis exposed that tensile testing caused the fibres to deform, leading to the formation of nodes [223,224].

In another study, Bıyık & Çoban [225] studied the potential of a bacterial strain isolated from a wine sample called *Acetobacter pasteurianus* for cellulose production using industrial waste and examined its performance with different carbon and nitrogen sources. The results showed that the presence of glucose and yeast extract in the media manufactured the highest quantity of microbial cellulose of 0.45 g/L. Among the industrial wastes (CSL, molasses, and whey), molasses produced the highest amount of BC (0.31 g/L). Further analysis of the structural properties of cellulose using Thin Layer Chromatography (TLC), Scanning Electron Microscopy (SEM), and Carbon-13 NMR revealed similarities in the structural characteristics of the BC with plant cellulose, indicated by the presence of non-branched polymer with D-glucopyranose units bonded with β-1, 4 bonds. Moreover, Voon et al. [226] used *Beijerinkia fluminensis* WAUPM53 and *Gluconacetobacter xylinus* 0416 bacteria to produce BC in sago byproducts (SBM), coconut water (CWM), and the standard Hestrin–Schramm mediums (HSM). The highest BC production was recorded in HSM followed by SBM and CWM for about 0.52 g/L, 0.47 g/L, and 0.45 g/L, respectively [226].

### 5.1. Brewery and Beverages Industries Wastes

There is a rising interest in the production of brewery and beverage industries because of the increasing user demand worldwide. According to Uzuner et al. [227], the beverage industry has become one of the biggest food processing industries. This industry can be categorized into two main groups that are non-alcoholic (i.e., whey, tea, cordial, coffee, apple, lassi, carbonated soft drink, etc.) and alcoholic drinks (i.e., whiskey, wine, beer, etc.). Carbonated soft drinks are consumed the most compared to other drinks, which are consumed at a rate of 48.8 gal/person, followed by bottled water, coffee, and beer with a value of 29.1, 24.6, and 21.8 gal/person, respectively [228]. This industry produces a large volume of waste per day and becomes a concern for management, spurring an effort to reduce the cost of disposal. These wastes are rich in nutrients; thus, they can be used for the biological treatment to produce BC for cost-effective and efficient waste management. Table 3 shows the evaluation of several waste or byproducts generated from the brewery and beverage industries to be used for BC production.

Whey is known to be rich in various nutritional components; hence, a growing literature body examines the feasibility of utilizing waste products as low-cost substrates for improved BC production [229]. Specifically, whey protein functions as an excellent source of nutrients. Revin et al. [230] examined the utilization of the dairy and alcohol industries acidic wastes, stillage (TS) and cheese whey, for the economical manufacturing of BC with *Gluconacetobacter sucrofermentans*. The findings revealed that, in three days of cultivation, the bacterial strain in whey produced up to 5.45 g/L of B and C structural properties analysis showed similarities between the synthesised cellulose with plant cellulose, despite morphological differences associated with crystallinity. The findings also indicated that acidic byproducts of dairy industries, such as wheat stillage and whey, are potential affordable sources of nitrogen and carbon for BC production.

Thin stillage (TS), a liquid byproduct produced after microbial fermentation of carbohydrates by yeast, contains various organic compounds. Hence, it is a potential source of nitrogen and carbon for BC synthesis. TS quantification via NMR methods showed that whey TS is rich in nutrition, containing high concentrations of lactic acid (7.41 g/L), dextrin (11.65 g/L), ethanol (1.31 g/L), acetic acid (2.72 g/L), and glycerol (7.87 g/L) [231]. TS wastewater from rice wine distilleries demonstrated the capability of producing BC with a concentration of 6.26 g/L in a seven days period of *Gluconacetobacter xylinus* cultivation [232]. From the study, it is confirmed that low-cost production of BC using TS as a substitute for HS medium is possible and the best alternative. Furthermore, the research revealed a facile and more practical approach for wastewater disposal. There have been efforts to enhance BC formation under static conditions by evaluating BC formation using *Gluconacetobacter xylinus* and a combination of whey and fruits as a culture medium by Jozala et al. [233]. The findings were in good correspondence to results achieved in other studies using *Gluconacetobacter sucrofermentans* [151].

### 5.2. Agro-Industry Waste

Several studies on the practicability of using different sources of agro-industry waste in BC production are reported. For instance, using *Komagataeibacter hansenii* for BC manufacturing from sisal juice as the substrate [234]. The researchers evaluated the effects of various variables on the potential of production, including the sugar concentration, pH, duration of cultivation, and nitrogen supplementation. From the findings, the best BC yield achieved from sisal waste was 3.38 g/L, which was yielded after 10 days of cultivation at a pH of 5. The study recommended that sisal waste is a precious resource for BC production; however, concerns arise regarding the ease of availability of sisal waste for large-scale manufacturing.

In a related study, Castro et al. [235] characterized the structural properties of BC obtained from agrochemical wastes of sugarcane and pineapple using *Gluconacetobacter swingsii*. HS medium was used as the reference standard for the comparisons. The results revealed that pineapple peel juice produced BC of higher quality than the reference standard, with values of 2.8 g/L and 2.1 g/L, respectively. The findings were parallel with other studies that concluded that utilizing agro-industry waste in general, and pineapple and sugarcane substrates in particular, are feasible for BC production. Whereas, when HS medium was utilized, some structural similarities were observed using SEM, while ATR-FR-IR spectra displayed chemical similarities in the microfibrils.

Zhao et al. [236] evaluated the potential of using yeast lees from fermentation vessels during fruit production using *Glucoacetobacter xylinum* for BC production. From the findings, yeast residue was identified as a potential substrate for economic BC production. However, for optimum production, modifications to the medium component and culture conditions of the bacterial strain are necessary. This is particularly important, given that the BC yield decreased with loading volume into cultivation vessels, which could have been associated with a reduced concentration of oxygen in the media [237].

For the yeast lees, researchers determined that mango pulp could supply essential substrate during BC production. Mango and guava purees displayed similar results due to the significant increase in water vapor permeability of the product [238]. Additional alterations in the produced BC included enhanced elongation and tensile strength reduction. Several studies suggested the addition of hydrophobic compounds [239] as a method to improve water resistance through cross-linking mechanisms [240].

Recent attempts to manufacture BC by *Gluconacetobacter xylinus* using pulp mill and lignocellulosic biorefinery waste fibre sludge displayed the potential to generate close to 11 g/L cellulose [241]. Producing high-quality BC at a low cost by utilizing sequential fermentation of residual streams from pulp mills and biorefinery processes is the most crucial contribution of this paper. The findings are in good agreement with the results achieved in other studies using various substrates.

There have been efforts to evaluate the possibility of utilizing other agricultural wastes for carbon sources in BC production, including corn products, coffee cherry husk (CCH), date fruits, and banana peel. CCH waste is an abundant agro-industrial waste. This method, using CCH as a substrate to produce BC achieved up to 8.2 g/L of BC using 8% of CCH extract combined with steep corn liquor under optimized conditions [242]. The findings were parallel with research evidence that steep corn liquor is rich in nutrition, which supplied organic content during BC production, such as carbon and nitrogen [243,244].

Banana peel is another potential agricultural waste being studied for a carbon source in BC production using *Acetobacter xylinum* [245]. The concentration of BC produced was 19.46 g/L of the product in a period of 15 days and a temperature of 30 ^o^C. Similar results were achieved with coconut water and pineapple juice as substrates for the same bacteria [245]. The date is a fruit with a potential carbon source for BC production. Date trees are grown mainly in tropical and arid areas of North Africa and Southwest Asia. Date syrup consists of essential nutrients that are sufficient for the growth of numerous microorganisms [246]. However, date processing is accompanied by massive loss and wastage, which can be converted to useful byproducts. Lotfiman et al. [247] assessed the viability of producing BC from date syrup using *A. Xylinum*. The researchers examined sugar content in the waste sample using HPLC and tested different concentrations of the fruit syrup at different culture times. BC production was attained with 3% (*w*/*v*) date in the medium cultivated for a duration of eight days. Alteration of the HS medium resulted in an increase in BC yield of up to 68%. It was determined that date waste is a potential source of carbon. Other cellulosic non-food wastes have also been utilized for BC formation with reduced BC yields, such as olive mill residues that produced 0.81 g/L of BC [248]. This BC yield was lower compared to date syrup. The findings indicated that agricultural waste could be used as a potential carbon source substitute compared to non-food sources.

Besides the substrates explained above, date industry waste is another possible substrate for the economic production of BC. One such byproduct of the industry is date syrup (DS), which is rich in carbohydrates [249]. A study utilized low-quality DS with very little commercial value; Moosavi-Nasab and Yousefi [246] found that BC production displayed a steady increment up to day 14 compared to sucrose, for which the production of BC remained almost constant. At the end of the cultivations, cellulose yield from DS (4.35 g/L) were more than two-folds that of sucrose (1.69 g/L). This is associated with the DS consisting of reducing sugars in abundance compared to sucrose, a disaccharide [246]. The same substrate was studied by Lotfiman et al. (2018) for the investigation of BC production by *A. xylinum.* Results of their study showed that *A. xylinum* produced up to 5.8 g/L of BC that was 68% higher compared to that of the standard HS medium [247].

Coffee cherry husk (CCH), a byproduct that is present in abundance from coffee cherry processing, is seen as a potential substrate for BC production [242]. Results of research attempts for BC production from CCH showed that the production capacity of up to 8.2 g/L was attained using 8% CCH extract combined with steep corn liquor under optimized conditions.

Besides the examples listed, banana peel is also being studied as a potential substrate for the economic production of BC. *Acetobacter xylinum* generated 19.46 g/L of BC in a cultivation period of 15 days at 30 °C [245]. Similar results were achieved when coconut water and pineapple juice were used as a substrate for the same microorganism [250].

### 5.3. Wastewater Sugar Industries, Pulp Mills and Lignocellulosic Biorefineries Wastes

Zhao et al. [236] studied the use of fermented wastewater as a substrate that showed a BC yield of 1.177 g/L, which much lower than HS medium (1.757 g/L). This could be ascribed to a low concentration of nitrogen and carbon contents in the substrate. However, BC yield from wastewater was sufficient to maintain large-scale commercial applications, with the low costs of the production process taken into consideration. These results are in good agreement with the existing evidence, which indicated that the ideal system for cellulose biosynthesis does not exist, even with gram-negative bacterium such as *Gluconacetobacter xylinus* that is able to secrete large volumes of cellulose as microfibrils from different waste products [251]. Other cellulosic wastes from non-food wastes are also being studied and have resulted in reduced BC yields [248].

In another related study, Li et al. [252] determined that jujube processing industry wastewater could provide an inexpensive raw material for BC production using *Gluconacetobacter xylinum* [252]. The experiments exhibited the potential to yield 2.25 g/L of BC in hydrolysate with acid treatment. However, the setup involves the usage of special filters of between 3 and 14 nm to produce nanostructures. This study showed the possibility of improving BC yield by adjusting the level of crystallinity and manipulating ammonium citrate concentration. Further research revealed that candied jujube consists of various nutritional compounds, such as amino acids, saccharides, and vitamins, which make an ideal substrate for BC synthesis of considerable quantities [253]. However, the crystallinity of the microbial cellulose is an important factor to be taken into consideration when utilizing jujube for BC, which was altered significantly in different cultivation media as a result of the effect of fibre size distribution. This effect is also observed during BC production using other waste feedstocks [254].

### 5.4. Textile Industries Waste

The growth of industrialization worldwide is affected by the increasing number of the world population. This phenomenon has resulted in an increase in the utilization of fabric and textiles to produce clothes and other materials related to textile-based products [90]. The growing demand for these products has led to the generation of tons of waste produced by the textile industries and consumers. Natural fibre resources such as cotton are commonly used in the textile industry to produce the fabric. According to Estur [255], world textile fibre consumption is projected to expand at an annual average rate of 4% to reach 70 million tons in 2010 and by 2.8% per year to reach 87 million tons in 2020. Used cotton fabric is not recycled because it does not provide a satisfactory level of use. It is usually dumped at the landfilled or garbage collection station or disposed of by incineration.

These waste cotton textiles have the potential to be used as an effective alternative to producing high-value products at low cost through enzymatic hydrolysis and microbial conversion processes. In addition, these wastes also have the potential to reduce environmental problems and save natural resources. Kuo et al. [256] conducted an experiment on enzymatic saccharification of dissolution pretreated cellulosic waste fabrics for BC production by *Gluconacetobacter xylinus*, which has shown that the BC produced from discoloured hydrolysate (1.88 g/L) by *G. xylinus* in static cultivation of seven days was about 20% higher compared to that in the coloured hydrolosate (1.59 g/L). This might be attributed to the fact that the coloured reducing sugars that were removed by chitosan adsorption prevent the fermentation activity of *Gluconacetobacter xylinus* for BC production.

Guo et al. [257] showed that BC could be successfully produced using waste dyed cotton fabrics cellulose through pretreatment with the ionic liquid (IL) 1-allyl-3-methyl-imidazolium chloride ([AMIM]Cl) with *Gluconacetobacter xylinus* followed by the production of enzymes with *Trichoderma reesei* via enzymatic saccharification. They found that the BNC yield obtained from the purple bed sheet (14.2 g/L) by *Gluconacetobacter xylinus* in static cultivation of 10 days was higher compared to that in the red bed sheet (13.7 g/L) and green bed sheet (14.1 g/L) [257]. Moreover, according to Guo et al. [257], this is due to the supplementation of calcium ions during treatment of Ca(OH)_2_ detoxification as well as the removal of dyes from the enzymatic hydrolysates.

Previous studies by Hong et al. [215] reported on production of high-quality carbon sources for BC from cotton-based waste textiles by *Gluconacetobacter xylinus.* These fabrics were pre-treated with the ([AMIM]Cl) followed by enzymatic hydrolysis. The results show that the yield and tensile strength of BC are 83% (10.8 g/L) and 79% (0.07 MPa) higher compared to a culture grown on a glucose-based medium [215]. The studies describing the use of textile mills waste for BC production are displayed in Table 3.

### 5.5. Biodiesel Industry Waste

BC is well known as a natural biomaterial with a broad range of applications. However, high-cost production in terms of raw materials, as well as low yields, have limited the industrial and commercial applications of BC. Hence, the usage of low cost-alternative raw materials as fermentation media would enhance BC production’s cost-competitiveness. The worldwide biodiesel production was more than 2.8 billion liters in 2018, and it has increased by 933% over the last 20 years [258,259]. It is estimated that crude glycerol is generated as a 10% (*w*/*w*) byproduct from transesterification of triglycerides with alcohol, most frequently methanol, which is equivalent to 0.28 billion liters.

In one of the studies, Tsouko et al. [259] investigated the feasibility of using fermentation media obtained from the confectionery industry and sunflower-based biodiesel industries waste streams and found that confectionary industry waste provides rich sources for carbon and nitrogen required for highly efficient BC production [259]. Batch fermentations using *Komagataeibacter sucrofermentans* (DSM) in synthetic media yielded BC concentrations of up to 13.3 g/L. The experimental results showed similar yields using both waste streams. The findings determined the significance of *Komagataeibacter sucrofermentans* DSM strain for high concentrations of BC production from the confectionery and biodiesel industry wastes. More importantly, the findings of this study on the water holding capacity (WHC) of the BC were parallel with the existing literature [260,261,262,263].

Previous studies reported the production and characterization of BC produced from non-detoxified crude glycerol as an alternative medium by *Gluconacetobacter xylinus* strain [264]. The highest BC production is 12.31 g/L. However, increasing crude glycerol has resulted in decreased BC production. This phenomenon might be due to the impurities in crude glycerol that might affect the activity of the cell. Besides that, from the research conducted by Soemphol et al. [264], it was shown that production of BC could improve by the addition of pineapple peel extract (PPE) into crude glycerol without any supplementation, and the optimal BC production was seen at acidic pH. The usage of these wastes or byproducts from biodiesel industries will not only produce value-added materials, it will also reduce environmental pollution and non-renewable energy consumption. The studies describing the use of biodiesel wastes for the production of BC are displayed in Table 3.

### 5.6. Micro-Algae Biomass Industries

Bioactive compounds such as carotenoids, polyunsaturated fatty acids, protein, vitamins, and minerals can be found in various commercial forms of micro-algal biomass (i.e., capsule, tablet, oil, liquid, flour, or powder forms). They play essential roles in numerous applications such as cosmetic products, pharmaceutical chemicals, feed product for animals (for fish, shellfish, poultry, and cattle) or functional food (i.e., supplements, dye, oil-derivatives, pastas, dairy products, and dessert) or with favorable outcomes upon human health, including antiviral, antimicrobial, anti-inflammatory, and antioxidant effects, as well as prevention of hypertension, diabetes, anaemia, constipation, and gastric ulcers [265]. Starch is one of the valuable constituents of microalgae biomass. Low-cost starch biomass products can be yielded from outdoor photobioreactors of *Chlorella cultures* microalgae [177]. Besides that, there are several studies that have been conducted to increase the starch content of algal biomass under different conditions (i.e., light intensities, nitrogen starvation, and sulphur). Freshwater algae *Chlorella vulgaris* can produce low-cost starch in large quantities (Dragone et al., 2011). This starch can be utilized as a promising alternative carbon source medium for the production of BC. Several studies reported the use of a byproduct of the micro-algae medium as a carbon source for BC. Several byproducts of micro-algae industries have been evaluated for BC production, as stated in Table 3.

Uzyol & Saçan [177] produced BC with *Komagataeibacter hansenii* using algae-based glucose, and showed that the BC production yields were 1.202 g/L and 1.104 g/L from glucose and algae-based glucose, respectively. The morphological structure of algae-based BC was observed to be similar to those of glucose-based BC. Another study, conducted on the production of green BC by utilizing renewable resources of algae with corn steep liquor [266], shows that the maximum BC production is 4.86 g/L. Therefore, based on the literature review, it can be summarized that integrating the metabolic components in algal biomass (i.e., corn steep liquor, glucose, yeast, starch, peptone, etc.) in the production of BC with the biorefinery concept would bring economic and environmental benefits, including the achievement of large scale production at low cost, and protecting the environment.

**Table 3:** Industrial wastes utilized as sustainable feedstock for the production of bacterial cellulose (BC).

Microorganism	Production Mode	BC Production	Time	Industrial Waste	Additional Nutrients	References
**Beverages/Brewery**						
**Waste as carbon source with additional nutrients**						
*Komagataeibacter xylinus* CICC No.10529	Static	5.7 g/L	8 days	Citrus peel and pomace enzymolysis medium	Yeast extract, ethanol and peptone	Fan et al. [216]
*Gluconacetobacter xylinus* NRRL B-42	Static	8.00 g/L	14 days	Grape bagasse	Corn steep liquor	Vazquez et al. [190]
*Gluconacetobacter xylinus* NRRL B-42	Static	7.20 g/L	14 days	Grape bagasse	Diammonium phosphate	
*Gluconacetobacter xylinus* ATCC^®®^ 10788™	Static	0.35 g/L	3 days	Makgeolli sludge filtrate	Modified HS (MHS) medium	Hyun et al. [267]
*Gluconacetobacter xylinus* ATCC^®®^ 10788™	Static	1.2 g/L	3 days	Makgeolli sludge filtrate	Mixed modified HS (MMHS)	
*Gluconacetobacter xylinus* BCRC 12334	Static	0.90 g/L	7 days	Thin stillage (TS) wastewater	50% TS	Wu & Liu [232]
*Gluconacetobacter xylinus* BCRC 12334	Static	6.26 g/L	7 days	Thin stillage (TS) wastewater	50/50 TS-HS	
*Gluconacetobacter oboediens*	Shaking	10.8 g/L	72 h	Distillery effluent	Sucrose (carbon source) and corn steep liquor (nitrogen source)	Jahan et al. [268]
*Gluconacetobacter hansenii* PJK KCTC 10505BP	Static	13.95 g/L	336 h	Untreated WBFB	1% Glucose	Ha et al. [269]
*Gluconacetobacter hansenii* PJK KCTC 10505BP	Shaking	1.50 g/L	168 h	Untreated WBFB	1% Glucose	
*Gluconacetobacter hansenii* PJK KCTC 10505BP	Static	7.37 g/L	336 h	Autolyzed WBFB	Glucose	
*Gluconacetobacter hansenii* PJK KCTC 10505BP	Static	3.64 g/L	336 h	Hydrolysed WBFB	1% Glucose	
**Waste as a complex medium without any additional nutrients**						
*Komagataeibacter saccharivorans strain BC1* *(K. saccharivorans strain BC1)*	Static	1.24 g/L	8 days	UB breweries limited, Baikampady, Mangalore, India	-	Gayathri et al. [270]
*Gluconacetobacter xylinus* BCRC 12334	Static	3.10 g/L	7 days	Thin stillage (TS) wastewater	-	Wu & Liu [232]
*Gluconacetobacter xylinus* NRRL B-42	Static	4.20 g/L	14 days	Grape bagasse	-	Vazquez et al. [190]
*Gluconacetobacter xylinus* ATCC^®®^ 10788™	Static	0.30 g/L	3 days	Makgeolli sludge filtrate	-	Hyun et al. [267]
*Gluconacetobacter medellinensis* ID13488	Static	1.5 g/L	14 days	Fresh apple peel/ sugar cane ratio (*w*/*w*) (1/2.3)	-	Urbina et al. [271]
*Gluconacetobacter medellinensis* ID13488	Static	1.4 g/L	14 days	Apple residue (AR)/ sugar cane (SC) ratio (*w*/*w*) (1/2.3)	-	
*Gluconacetobacter medellinensis* ID13488	Static	2.0 g/L	14 days	AR/SC ratio (*w*/*w*) (0.5/2.8)	-	
*Gluconacetobacter medellinensis* ID13488	Static	1.2 g/L	14 days	AR/SC ratio (*w*/*w*) (2/1.3)	-	
*Gluconacetobacter medellinensis* ID13488	Static	2.5 g/L	14 days	AR/SC ratio (*w*/*w*) (1.5/2.3)	-	
*Gluconoacetobacter xylinum* ATCC 23768	Static	2.9 g/L	9 days	Black strap molasses	-	Khattak et al. [272]
*Gluconoacetobacter xylinum* ATCC 23768	Shaking	3.05 g/L	9 days	Black strap molasses	-	
*Gluconoacetobacter xylinum* ATCC 23768	Static	1.70 g/L	9 days	Brewery molasses	-	
*Gluconoacetobacter xylinum* ATCC 23768	Shaking	1.75 g/L	9 days	Brewery molasses	-	
*Gluconacetobacter oboediens*	Shaking	8.5 g/L	72 h	Crude effluent	-	Jahan et al. [268,273]
*Acetobacter xylinum* NRRL B-42	Static	6.7 g/L	21 days	Grape pomace extract/corn steep liquor	-	Cerrutti et al. [274]
*Gluconacetobacter hansenii* PJK KCTC 10505BP	Static	8.46 g/L	336 h	Untreated Waste from beer fermentation broth (WBFB)	-	Ha et al. [270]
*Gluconacetobacter hansenii* PJK KCTC 10505BP	Static	2.00 g/L	336 h	Autolyzed WBFB	-	
*Gluconacetobacter hansenii* PJK KCTC 10505BP	Static	2.82 g/L	336 h	Hydrolysed WBFB	-	
*Gluconacetobacter sucrofermentans B*-*11267*	Shaking	2.40 g/L	3 days	Hestrin and Schramm (HS) medium	-	Revin et al. [230]
*Gluconacetobacter sucrofermentans B*-*11267*	Shaking	6.19 g/L	3 days	Thin stillage	-	
*Gluconacetobacter sucrofermentans B*-*11267*	Shaking	5.50 g/L	3 days	Cheese whey	-	
*Gluconacetobacter sucrofermentans B*-*11267*	Shaking	6.19 g/L	3 days	Thin stillage pH 3.95	-	
*Gluconacetobacter sucrofermentans B*-*11267*	Shaking	5.40 g/L	3 days	Thin stillage pH 5	-	
*Gluconacetobacter sucrofermentans B*-*11267*	Shaking	3.50 g/L	3 days	Thin stillage pH 6	-	
*Gluconacetobacter xylinus*	Static	2.90 g/L	4 days	Acid hydrolysate of waste oleaginous yeast biomass	-	Luo et al. [275]
*Gluconacetobacterhansenii* CGMCC 3917	Static	3.89 g/L	14 days	Waste beer yeast treated with ultrasonication treatment	-	Lin et al. [237]
*Gluconacetobacterhansenii* CGMCC 3917	Static	2.40 g/L	14 days	Waste beer yeast treated with NaOH treatment	-	
*Gluconacetobacterhansenii* CGMCC 3917	Static	2.00 g/L	14 days	Waste beer yeast treated with high speed homogenizer treatment	-	
*Gluconacetobacterhansenii* CGMCC 3917	Static	1.50 g/L	14 days	Waste beer yeast treated with microwaves treatment	-	
*Gluconacetobacterhansenii* CGMCC 3917	Static	1.20 g/L	14 days	Waste beer yeast treated with untreatment	-	
*Gluconacetobacter* *xylinus BC-11 K.*	Static	1.18 g/L	10 days	Wastewater after pullulan polysaccharide fermentation	-	Zhao et al. [236]
**Agro industrial waste**						
**Waste as nitrogen source**						
*Gluconacetobacter swingsii*	Static	2.8 g/L	13 days	Pineapple peel juice	Glucose, fructose and sucrose	Castro et al. [235]
**Waste as carbon source with additional nutrients**						
*Gluconacetobacter swingsii*	Static	-	13 days	Sugar cane juice	Glucose, fructose and sucrose	Castro et al. [235]
*Gluconacetobacter xylinum bacterium (ATCC 700178)*	Shaking	10.6 g/L	7 days	Wheat straw	Corn steep liquor (CSL)	Goyat [266]
*Gluconacetobacter* *xylinus*	Static	1.8 g/L	9 days	Carob and haricot bean (CHb) medium	Citric acid	Bilgi et al. [276,277]
*Komagataeibacter rhaeticus*	Static	6.0 g/L	7 days	HS medium and Cashew tree exudates (HSCTE)	HS medium	Pacheco et al. [278]
*Komagataeibacter rhaeticus*	Static	6.0 g/L	7 days	HS medium and Cashew tree exudates (HSCG)	HS medium	
*Acetobacter aceti* ATCC 23770	Shaking and static	2.12 g/L	8 days	Cheap agricultural product konjac powder	Yeast extract and tryptone	Hong & Qiu [279]
*Gluconacetobacter hansenii* UAC09	Static	8.2 g/L	14 days	Coffee cherry husk (CCH)	8% corn steep liquor (CSL)	Rani & Appaiah [242]
*Gluconacetobacter hansenii* UAC09	Static	6.5 g/L	14 days	Coffee cherry husk (CCH)	0.2% Urea	
*Gluconacetobacter hansenii* UAC09	Static	6.9 g/L	14 days	Coffee cherry husk (CCH)	Ethyl alcohol (EA) + Acetic acid (AA)	
*Gluconacetobacter hansenii* UAC09	Static	7.5 g/L	14 days	Coffee cherry husk (CCH)	8% CSL + EA + AA	
*Gluconacetobacter hansenii* UAC09	Static	6.6 g/L	14 days	Coffee cherry husk (CCH)	0.2% urea + EA + AA	
*Acetobacter xylinus* ATCC 23770	Static	8.3 g/L	7 days	Enzymatic hydrolysate of wheat straw	Other components are same as of HS medium	Chen et al. [280]
*Acetobacter xylinum* 0416 MARDI	Static	4.0 g/L	8 days	Extracted date syrup (DSH-2%)	Other components are same as of HS medium	Lotfiman et al. [247]
*Acetobacter xylinum* 0416 MARDI	Static	5.8 g/L	8 days	Extracted date syrup (DSH-3%)	Other components are same as of HS medium	
*Acetobacter xylinum* 0416 MARDI	Static	4.5 g/L	8 days	Extracted date syrup (DSH-5%)	Other components are same as of HS medium	
*Gluconacetobacter sacchari*	Static	0.1 g/L	96 h	Grape skins aqueous extract, cheese whey, crude glycerol and sulfite pulping liquor	Organic or inorganic nitrogen	Carreira et al. [281]
*Acinetobacter sp. BAN1*	Static	0.3 g/L	15 days	Pineapple juice medium (PIJM)	Other components are same as that of HS medium	Adebayo-Tayo et al. [282]
*Acinetobacter sp. BAN1*	Static	6.4 g/L	15 days	Pawpaw juice medium (PAJM)	Other components are same as that of HS medium	
*Acinetobacter sp. BAN1*	Static	0.6 g/L	15 days	Watermelon juice medium (WMJM)	Other components are same as that of HS medium	
*Acetobacter pasteurianus PW1*	Static	0.1 g/L	15 days	Pineapple juice medium (PIJM)	Other components are same as that of HS medium	
*Acetobacter pasteurianus PW1*	Static	7.7 g/L	15 days	Pawpaw juice medium (PAJM)	Other components are same as that of HS medium	
*Acetobacter pasteurianus PW1*	Static	0.4 g/L	15 days	Watermelon juice medium (WMJM)	Other components are same as that of HS medium	
*Gluconoacetobacter xylinus* BCRC 12334	Static	3.40 g/L	8 days	Orange peel fluid and orange peel hydrolysate	Acetate buffer, peptone and yeast extract	Kuo et al. [214]
*Beijerinkia fluminensis* WAUPM53	Static	0.47 g/L	14 days	Sago byproduct	Other components are same as of HS medium	Voon et al. [226]
*Gluconacetobacter xylinus* 0416	Static	1.55 g/L	14 days	Sago byproduct	Other components are same as of HS medium	
*Acetobacterxylinum* NBRC 13693	Static	4.1 g/L	14 days	Pineapple	Disodium hydrogen phosphate buffer	Kurosumi et al. [283]
*Acetobacterxylinum* NBRC 13693	Static	3.95 g/L	14 days	Apple	Disodium hydrogen phosphate buffer	
*Acetobacterxylinum* NBRC 13693	Static	5.9 g/L	14 days	Orange	Disodium hydrogen phosphate buffer	
*Acetobacterxylinum* NBRC 13693	Static	3.5 g/L	14 days	Japanese pear	Disodium hydrogen phosphate buffer	
*Acetobacterxylinum* NBRC 13693	Static	1.8 g/L	14 days	Grape	Disodium hydrogen phosphate buffer	
*Acetobacterxylinum* NBRC 13693	Static	0.5 g/L	14 days	Pineapple	Sugar reagent (glucose, fructose and sucrose)	
*Acetobacterxylinum* NBRC 13693	Static	0.2 g/L	14 days	Apple	Sugar reagent (glucose, fructose and sucrose)	
*Acetobacterxylinum* NBRC 13693	Static	1.85 g/L	14 days	Orange	Sugar reagent (glucose, fructose and sucrose)	
*Acetobacterxylinum* NBRC 13693	Static	0.5 g/L	14 days	Japanese pear	Sugar reagent (glucose, fructose and sucrose)	
*Acetobacterxylinum* NBRC 13693	Static	0.4 g/L	14 days	Grape	Sugar reagent (glucose, fructose and sucrose)	
*Gluconacetobacter sacchari*	Static	1.7 g/L	96 h	Dry olive mill residue (DOR100) Water extraction at 100 °C	Nitrogen	Gomes et al. [248]
*Gluconacetobacter sacchari*	Static	1.4 g/L	96 h	Dry olive mill residue (DOR100) Water extraction at 100 °C	Phosphorus	
*Komagataeibacter hansenii* MCM B-967	Static	125 g/L	7 days	Pineapple and watermelon peels	Sucrose, ammonium sulfate and cycloheximide	Kumbhar et al. [284]
*Acetobacter xylinum* DSMZ2004	Static	8.6 g/L	48 h	Poor quality apple residues in combination with glycerol	Apple glucose equivalents, glycerol, ammonium sulfate and citric acid	Casarica et al. [285]
*Acetobacter xylinum BCRC 14182 (purchased)*	Static	-	3–7 days	Coconut-water	Sugar	Lin et al. [286]
**Waste as complex medium without any additional nutrients**						
*Komagataeibacter hansenii* GA2016	Static	2.06 BC/100 g peel	21 days	Lemon peels (LBC)	-	Güzel & Akpınar [287]
*Komagataeibacter hansenii* GA2016	Static	3.92 BC/100 g peel	21 days	Mandarin peels (MBC)	-	
*Komagataeibacter hansenii* GA2016	Static	2.33 BC/100 g peel	21 days	Orange peels (OBC)	-	
*Komagataeibacter hansenii* GA2016	Static	2.68 BC/100 g peel	21 days	Grapefruit peels (GBC)	-	
*Komagataeibacter xylinus*	Static	2.90 g/L	10 days	Discarded waste durian shell	-	Luo, Huang et al. [275]
*Gluconacetobacter xylinus* CH001	Static	2.67 g/L	10 days	Discarded waste durian shell	-	Luo, Huang, et al.[288]
*Gluconacetobacter* *medellinensis*	Static	3.24 g/L	7 days	Sugar cane juice and pineapple residues	-	Algar et al. [289]
*Gluconacetobacter* *medellinensis*	Dynamic	0.82 g/L	7 days	Sugar cane juice and pineapple residues	-	
*Acinetobacter* sp. BAN1	Static	0.4–0.6 g/L	15 days	Pineapple waste medium (PIWAM)	-	Adebayo-Tayo et al. [290]
*Acinetobacter* sp. BAN1	Static	0.2–1.1 g/L	15 days	Pawpaw waste medium (PAWAM)	-	
*Acetobacterpasteurianus* PW1	Static	0.2–1.0 g/L	15 days	Pawpaw waste medium (PAWAM)	-	
*Acetobacterpasteurianus* PW1	Static	0.1–3.9 g/L	15 days	Pineapple waste medium (PIWAM)	-	
*Komagataeibacter rhaeticus* iGEM	Static	–	10 days	Fermented tea	-	Florea et al. [291]
*Gluconacetobacter* *sacchari*	-	1.28 g/L	-	Industrial residues from olive oil production	-	Gomes et al. [248]
*Gluconacetobacterpersimmonis* GH-2	Static	5.75 g/L	14 days	Watermelon + HS medium	-	Hungund et al. [292]
*Gluconacetobacterpersimmonis* GH-2	Static	5.98 g/L	14 days	Orange juice + HS medium	-	
*Gluconacetobacterpersimmonis* GH-2	Static	6.18 g/L	14 days	Muskmelon + HS medium	-	
*Gluconacetobacterpersimmonis* GH-2	Static	8.08 g/L	14 days	Coconut water +HS medium	-	
*Acetobacter* *xylinum*	Static	19.46 g/L	15 days	Banana peel	-	Hungund et al. [245]
*Gluconacetobacterxylinus* ATCC 53582	Static	60 g/L	96 h	Rotten fruit culture	-	Jozala et al. [293]
*Gluconacetobacterxylinus* CGMCC 2955	Static	2.25 g/L	114 h	Waste water of candied jujube hydrolysate	-	Li et al. [252]
*Acetobacterxylinum* 0416	Rotary disc reactor	28.30 g/L	4 days	Pineapple waste medium	-	Zahan et al. [197]
*Komagataeibacter rhaeticus*	Static	2.8 g/L	7 days	Cashew tree exudates (CTE)	-	Pacheco et al. [278]
*Komagataeibacter rhaeticus*	Static	2.3 g/L	7 days	Cashew gum (CG)	-	
*Gluconacetobacter hansenii* UAC09	Static	5.6 g/L	14 days	Coffee cherry husk (CCH)	-	Rani & Appaiah [242]
*Gluconacetobacter sacchari*	Static	0.81 g/L	96 h	Dry olive mill residue (DOR40) Water extraction at 40 °C	-	Gomes et al. [248]
*Gluconacetobacter sacchari*	Static	0.85 g/L	96 h	Dry olive mill residue (DOR100) Water extraction at 100 °C	-	
**Sugar industries, pulp mills and lignocellulosic biorefineries wastes**						
**Waste as carbon source with additional nutrients**						
*Komagatacibacter xylinus PTCC 1734*	Static	7.02 g/L	10 days	Vinasse	Other components are same as of HS medium	Barshan et al. [294]
*Acetobacter xylinum* BPR2001	Rotary shaker	3.01 g/L	70 h	Molasses	Corn steep liquor	Bae & Shoda [187]
*Acetobacter xylinum* BPR2001	Rotary shaker	5.30 g/L	70 h	H_2_SO_4_ heat treated molasses	Corn steep liquor	
*Gluconacetobacter xylinus*	Static	5.9 g/L	14 days	Cane molasses	Corn steep liquor and diammonium phosphate	Vazquez et al. [190]
*Acetobacter sp. V6*	Agitated	3.12 g/L	168 h	Molasses and corn steep liquor	Acetic acid	Jung et al. [204]
*Acetobacter xylinum* ATCC 10245	Static	223% as compared to 100% in HS medium	7 days	Sugar cane molasses	Carbohydrates, minerals, vitamins and amino acids	Premjet et al. [295]
*Komagataeibacter rhaeticus*	Static	3.90 g/L	120 h	Sugarcane molasses (SCM) 10 g/L of SCM	40 g/L of glucose	Machado et al. [296]
*Komagataeibacter rhaeticus*	Static	4.01 g/L	120 h	20 g/L of SCM	30 g/L of glucose	
*Komagataeibacter rhaeticus*	Static	3.7 g/L	120 h	30 g/L of SCM	20 g/L of glucose	
*Komagataeibacter rhaeticus*	Static	3.50 g/L	120 h	40 g/L of SCM	10 g/L of glucose	
*Gluconacetobacter xylinus* ATCC 23770	Static	11 g/L	7 days	Waste fiber sludge sulfate	Yeast extract and tryptone	Cavka et al. [241]
*Gluconacetobacter xylinus* ATCC 23770	Static	10 g/L	7 days	Waste fiber sludge sulfite	Yeast extract and tryptone	
*Acetobacter xylinum* ATCC 10245	Static	20.6 %	7 days	Softwood purified water-soluble (SPWS)	Other components are same as of HS medium	Uraki et al. [297]
*Acetobacter xylinum* ATCC 10245	Static	33 %	7 days	Hardwood purified water-soluble (HPWS)	Other components are same as of HS medium	
*Acetobacter xylinum* ATCC 53582	Static	5.4 %	7 days	Softwood purified water-soluble (SPWS)	Other components are same as of HS medium	
*Acetobacter xylinum* ATCC 53582	Static	8.9 %	7 days	Hardwood purified water-soluble (HPWS)	Other components are same as of HS medium	
**Waste as carbon source without any additional nutrients**						
*Acetobacter xylinus* 23769		0.15 g/L		Hot water extract	-	Erbas Kiziltas et al. [298]
*Gluconoacetobacter xylinum* ATCC 23768	Shaking	2.51 g/L	10 days	Scum of sugarcane jaggery or gur (JS)	-	Khattak, Khan, Ul-Islam, Wahid, et al. [299]
*Gluconoacetobacter xylinum* ATCC 23768	Static	2.13 g/L	10 days	Scum of sugarcane jaggery or gur (JS)	-	
*Komagataeibacter europaeus* SGP37	Static	6.30 g/L	16 days	Sweet lime pulp waste	-	Dubey et al. [300]
*G. persimmonis GH-2*	Static	5.75 g/L	14 days	Molasses + HS medium	-	Hungund et al. [292]
*G. intermedius SNT-1*	Static	12.6 g/L	10 days	Molasses pretreated with hea	-	Tyagi et al. [301]
*Gluconacetobacter xylinus (PTCC*, *1734)*	Static	4.35 g/L	336 h	Date syrup	-	Moosavi-Nasab [246]
*Komagataeibacter rhaeticus*	Static	1.90 g/L	120 h	50 g/L of SCM	-	Machado et al. [296]
*Gluconaceterxylinus* CH001	Static	0.66 g/L	5 days	Lipid fermentation wastewater	-	Huang et al. [302]
*Gluconaceter* *xylinus*	Static	1·34 g/L	7 days	Acetone-butanol-ethanol(ABE) fermentation wastewater	-	Huang et al. [303]
*Gluconaceter* *xylinus BC-11*	Static	1.177 g/L	10 days	Wastewater after pullulan polysaccharide fermentation	-	Zhao et al. [236]
*Acetobacterxylinum* 23769	Static	0.15 g/L	672 h	Wood hot water extract	-	Erbas Kiziltas et al. [298]
**Textile mills**						
**Waste as carbon source with additional nutrients**						
*Gluconacetobacter xylinus ATCC 23770*	Static	10.8	14 days	Cotton-based waste textiles	Glucose, yeast extract and peptone	Hong et al. [215]
*Gluconacetobacter xylinus*	Static	14.2 g/L	10 days	Waste dyed cotton fabrics hydrolysate - Purple bed sheet (PBS)	Peptone and yeast extract	Guo et al. [257]
*Gluconacetobacter xylinus*	Static	13.7 g/L	10 days	Waste dyed cotton fabrics hydrolysate- rose -Red bed sheet (RRBS)	Peptone and yeast extract	
*Gluconacetobacter xylinus*	Static	14.1 g/L	10 days	Waste dyed cotton fabrics hydrolysate- green bed sheet (GBS)	Peptone and yeast extract	
*Gluconacetobacter xylinus*	Static	1.59 g/L	7 days	Coloured hydrolysate	Peptone and yeast extract	Kuo et al. [256]
*Gluconacetobacter xylinus*	Static	1.88 g/L	7 days	Discoloured hydrolysate	Peptone and yeast extract	Kuo et al. [256]
**Biodiesel industry**						
**Waste as carbon source with additional nutrients**						
*Gluconaceter xylinus* BNKC19	Static	12.31 g/L	7 days	Non-detoxified crude glycerol	Pineapple and in combination with HS medium components	Soemphol et al. [264]
*Gluconacetobacter xylinus* DSM 46604	Agitated	2.87 g/L	5 days	20 g/L glycerol	Yeast extract, ammonium sulphate, potassium hydrogen orthophosphate and magnesium sulphate	Adnan [304]
*Gluconacetobacter xylinus* DSM 46604	Agitated	2.87 g/L	5 days	50 g/L glucose	Yeast extract, ammonium sulphate, potassium hydrogen orthophosphate and magnesium sulphate	Adnan [304]
*Gluconacetobacter xylinus*	Static	10 g/L	14 days	Glycerol from biodiesel	Diammonium phosphate and corn steep liquor	Vazquez et al. [190]
*Gluconacetobacter intermedius* NEDO-01	Static	3.4 g/L	4 days	Waste glycerol	Carboxymethyl Cellulose	Kose et al. [305]
*Komagataeibacter sucrofermentans DSM 15973*	Shaking	3.2 g/L	15 days	Crude glycerol from biodiesel	Yeast extract and peptone	Tsouko et al. [259]
*Komagataeibacter sucrofermentans DSM 15973*	Shaking	13.3 g/L	15 days	Crude glycerol from biodiesel	Sunflower meal hydrolysates	Tsouko et al. [259]
*Komagataeibacter sucrofermentans DSM 15973*	Shaking	13 g/L	15 days	Crude glycerol from biodiesel	Flour-rich hydrolysates	Tsouko et al. [259]
**Waste as carbon source without additional nutrients**						
*Gluconacetobacter xylinus*	Static	3.5 g/L	14 days	Glycerol from biodiesel	-	Vazquez et al. [190]
**Micro-algae industry**						
**Waste as carbon source with additional nutrients**						
*Gluconacetobacter xylinum bacterium (ATCC 700178)*	Shaking	4.86 g/L	7 days	Algae	Corn steep liquor (CSL)	Goyat [266]
*Gluconacetobacter xylinus (ATCC #700178)*	Static	77%	7 days	*Chlorella vulgaris*	Glucose/yeast extract	Chen et al. [306]
*Gluconacetobacter xylinus (ATCC #700178)*	Static	94%	7 days	*Scenedesmus obliqnus*	Glucose/yeast extract	
*Gluconacetobacter xylinus (ATCC #700178)*	Static	85%	7 days	*Chlamydomonas reinhardtii*	Glucose/yeast extract	
*Komagataeibacter hansenii* DSMZ	Static	1.104 g/L	7 days	Algae (*Chlorella vulgaris*) algae based glucose	Meat extract, peptone, NaCl and ethanol	Uzyol & Saçan [177]
**Waste as carbon source without additional nutrients**						
*Komagataeibacter saccharivorans*	Static	85.1%	14 days	Algae (*Chlamydomonas debaryana*) (BEA0067)	-	Nóbrega et al. [307]

## 6. Future Perspectives

Different industrial sectors produce a large amount of waste on a daily basis. More brewery, sugar, lignocellulosic, and other industrial wastes could be valorized as complex media without additional nutrient sources or as carbon and nitrogen sources with additional nutrient sources for BC production. Due to their large-scale availability and increased BC productivity, agro-industrial wastes can be widely utilised for BC production. Due to increased urbanization around the world, particularly in economically developing nations, municipal waste is anticipated to become an increasingly major source of waste biomass with higher organic content. All of the low-cost waste media of the industries discussed here, especially those that do not require complex pre-treatments, detoxification, or supplementing, have a lot of potential for upscale production of BC on an industrial scale. When compared to regular media, BC created from waste media has similar physico-chemical characteristics and a higher yield.

Because these wastes are available in huge quantities, waste producers may be able to sell them to commercial BC producers or academic institutions. The BC that was obtained could be used as a raw material by a variety of biomedical enterprises for commercial purposes as well as by scientists for study. Since BC derived from some agro-wastes might be colored and absorb unwanted compounds, proper purification is required. These facts may justify limiting the use of BC in industries with stringent regulatory standards, such as biomedicine, pharmaceutics, cosmetics, or the food industry. In terms of the environment, eliminating these industrial outputs will allow for proper waste management, lowering the environmental and health risks associated with these wastes. This will be a realistic option for dealing with pollution issues.

## 7. Conclusions

Bacterial cellulose (BC) is considered a desirable biomaterial for various applications across many fields due to its unique structural features and desirable properties. This review mainly discusses the technical and economic feasibility of producing microbial cellulose from industrial wastes from agro-industry, textile, biodiesel, micro-algae biomass, wastewater sugar, and lignocellulosic biorefineries, breweries, and beverages. The overarching conclusion is that most industrial wastes have the potential to produce high concentrations of BC. The production of high concentrations of BC can be obtained by optimizing bacterial culture conditions, such as temperature and pH. More importantly, the findings demonstrate that the produced microbial cellulose would have desirable chemical, physical, and mechanical properties, which suit various advanced applications. This review shows that the production of BC from industrial waste is successful. The future of using industrial wastes for BC production seems promising, since the source of nutrients in BC production from industrial wastes has reduced the production cost. Moreover, tonnes of industrial waste are generated daily, and using some of these wastes in BC production can mitigate waste disposal problems. The high yield and low production cost of BC is the main challenge that needs to be contemplated. A lot of progress can be made by developing new fermentation methods, new bioreactor design, and using a cheaper waste media that aims to increase the yield of BC at a lower cost. The BC has been used in various industries in manufacturing products as well as advanced applications. Products such as BC masks, BC gloves, paper, biodegradable food packaging, and wound dressing have been on the market. More advanced BC applications have shown promising results, such as never-dried microbial cellulose membranes, skin transplants, optically transparent cellulose nanocomposites, and artificial bacterial cellulose ligaments. Overall, large-scale commercial production and demand of microbial cellulose using waste as a carbon and energy source can lower the biomaterial production cost and help eliminate or reduce the economic and environmental burden of industrial waste.

## Figures and Tables

**Table 1:** Fabrication of BC and BC-based biocomposites under static and agitation culture methods, their properties, and applications.

Bacterial Cellulose and Bacterial Cellulose-Based Biocomposites	Applications	Structure and Properties	References
**Fabrication of BC and BC-based composites under static culture methods**			
**BC**	BC mask	Fast healing, high moisture donation, and high conformability	Saxena et al. [107]
	Blood vessel; Vascular grafts	Excellent mechanical properties, thin layers, dense	Putra et al. [108]
	Implant material for auricular cartilage regeneration and for ear cartilage replacement	Compatible mechanical strength and patient-specific shapes	Nimeskern et al. [109]
	Potential meniscus implant	High compression strain and mechanical strength	Bodin et al. [110]
	Replacement of blood vessels and diseased arteries	High water holding capacity and mechanical strength	Charpentier et al. [111]
	Artificial blood vessels for microsurgery	The smooth inner surface, moldability, and high mechanical properties	Klemm et al. [112]
	Artificial cornea and eye bioengineering Retinal pigment epithelium (RPE)	High elastic modulus, tensile strength and elongation at break, high initial cell adhesion, porous, permeable up to 300 kDa, and dimensionally stable	Padra et al. [98]
**BC/polycaprolactone biocomposites**	Tissue substitutes in rabbits’ cornea	Signs of the moderate inflammatory process, pro- tected ocular surface and remained stable in corneal tissue during the 45-day follow-up	Sepúlveda et al. [113]
**BC/polycaprolactone (PCL) biocomposites**	Biodegradable food packaging	Good transparency of the BC/PCL, smooth surface morphology	Barud et al. [114]
**BC/benzoyltrifluoroacetone**	Phosphors and UV to Visible energy converting devices	Improvement of the luminescence characteristics	Caiut et al. [115]
**BC/ AgNPs/ lginate-molybdenum trioxide nanoparticles (MoO_3_NPs)**	Hydrogen sulfide (H_2_S) gas sensor	Successfully detected H_2_S gas	Sukhavattanakul et al. [102]
**BC/chitosan biocomposites**	Wound dressing	The improved proliferation and fibroblast adhesion	Kim et al. [116]
**BC/Lipase nanocomposites**	Bioactive paper for developing a simple, handheld, and disposable devices; industrials bio- processes of detergents and food industry and biomedicine	Specific activity was higher for BC/ Lipase suspension (4.2 U/mg), improved thermal stability, reusability, and durability	Buruaga-Ramiro [78]
**BC/ SOD (Procel-Super) and poviargol (Procel-PA) biocomposites**	Skin regeneration scaffold; Membranes for skin tissue regeneration	Highly transparency, antibacterial activity	Legeza et al. [117]
**BC/ PVOH**	The food industry, food packaging	Improved mechanical properties; UV-light barrier properties; Reduce WVP and porosity value	Cazón et al. [3]
**BC/ PHB**	Food packaging applications	low dispersion of BC in the matrix; increased crystallinity of the incubated samples; low interfacial adhesion	Seoane et al. [99]
**BC/ciprofloxacin biocomposites**	Contact lens for better tissue regeneration, enhance the recovery of ocular burns, replacement for antibiotics eye drops, wound dressing after eye surgery or protection against bacteria.	No mutagenicity, genotoxicity and cytotoxicity effects	Messaddeq et al. [118]
**BC/ polyvinyl alcohol coated biochar nanosilver biocomposites**	Drinking water treatment	BC was uniformly mixed into the PVA gel; PVA/BC/C-Ag composite membranes exhibited excellent antibacterial activity; good reusability	Zhang et al. [100]
**BC/polycaprolactone biocomposites**	Tissue substitutes in rabbit cornea	High transparency and mechanical properties	Sepúlveda et al. [113]
**BC/polyvinyl alcohol biocomposites**	BC gloves	Skin cell support and fabrication of optimal moist condition	Osorio et al. [119]
**BC/ cAgNP**	Wound dressing	High cytocompatibility; high moisture content and; good level of transparency; broad-spectrum antimicrobial activity along with antioxidant properties	Gupta et al. [103]
**Fabrication of BC and BC-based composites under agitation/shaking culture method**			
**BC**	Sewage treatment; Immobilized reaction; Adsorption of Pb^2+^ bio-separation and bovine serum albumin	Porous and loose structure, BC adhering to each other; diameter of composites with a size range of 0.5–6 mm	Zhu, Jia, Yang, et al. [120]
	The production of immobilized glucoamylase was supported by BC spheres for industrial applications usage	BC spheres were produced with various range of sizes such as 0.5–1.5, 2–3, and 4–5 mm; Large functional group, as well as great surface area to connect with enzymes, resulted to the higher activity of small spheres.	Wu & Li, [121]
	For good viability and adhesion on the surface of the material	Sphere formation was affected by temperature; solid structure formed; diameter of composites with a size range of 2–8 mm formed	Hu et al. [122]
	Fermentation	IR: 6.52–3.85; Crystallinity: 81.43–84.35 %; Flocky asterisk-like; diameter of composites with a size range of 5–10 mm,	Bi et al. [24]
	Food, healthcare, and materials applications	Diameter is less than 1–8 mm at 150 rpm; Form solid structure however the central region is not layered; Layer spacing 10 μm (150 rpm) and 20 μm (125 rpm)	Hu & Catchmark [123]
	Good production yield	Thinner microfibrils structure; IR: 4.48; crystallinity: 84%; large and unique spheres; diameter of composites with a size range of 5–10 mm	Czaja et al. [124]
	High-efficiency lipase-immobilization system for large-scale industrial hydrolysis of fats and oils Suitable for enzymatic immobilization.	High hydrolytic activity; High operational activity; Lipase immobilized BC sphere; Size of diameter between 3–9 mm.	Cai et al. [125]
	Pectin and xyloglucan can be used to enhance cellulose growth and cellulose assembly.	Xyloglucan: Layered structure, densely packed cellulose bundles with the layered structure were formed; Central core is not obviously seen; diameter of composites with a size range of 4–5 mm; aster-likePectin: Densely packed cellulose bundles with layered structure; diameter of composites with a size range of 5–6 mm; aster-like Xylan: Pore structure of cellulose bundles with a few tails formed on the surface of sphere; diameter of composites with a size range of 7–8 mm; layered structure Arabinogalactan: Cellulose linkage between layered structure; diameter of composites with a size range of 4–6 mm; Sphere	Gu & Catchmark [126]
**BC/ schizophyllan (SPG) biopolymers**	Anti-wrinkle dressing masks, wound healing and absorption materials	Mechanical, swelling and antibacterial properties were improved; moderate antibacterial activity	Hamedi et al. [101]
**BC/CNT biocomposites**	--	BC: IR index 2.23, crystallinity 67.2%; snow like structuredBC/CNT composites: IR index 2.56; crystallinity 76.2%, the diameter of composites with a size range of 2–5 mm, rice-like structured	Yan et al. [127]
**BC/Fe_3_O_4_ biocomposites**	Elution: Mn^2+^ > Pb^2+^ > Cr^3+^ Superparamagnetic Adsorption: Pb^2+^ > Mn^2+^ > Cr^3+^	Iron(II,III) oxide (Fe_3_O_4_) particles with a size of 15 nm were distributed uniformly in spheres The diameter of composites with a size range of 3–5 mm	Zhu, Jia, Wan, et al. [128]
**BC/GO biocomposites**	Superabsorbent for water environmental protection	Superior absorption capacity; Interconnected structure with a honeycomb-like surface pattern; diameter of composites with a size range of 3–7 mm	Hu [129]

**Table 2:** Various modifications, product specifications, and advantages of different reactors for BC production.

Modification	Production Specification and Advantages	BC Production	References
**Enriched Oxygen Bioreactors**			
**Bubble column (controlled pH)** **Aeration rate:1.0 vvm (30 L/min)**	**Attributes:** Low concentrated solution state culture; Low shear stress; Low mechanical properties: 17.15 to 11.66 MPa; Low crystallinity: 86 to 79.6%, Low degree of polymerization and molecular weight **Advantages**: High oxygen transfer rate	0.07–0.09 g/L/h	Choi et al. [192]
**High oxygen concentration**	**Attribute:** After 30 h the production decreased **Advantages:** Higher productivity; High oxygen transfer rate; Low power requirement.	0.20 g/L/h	Chao et al. [193]
**Internal loop airlift with controlled pH/ fresh and glucose medium**	**Attribute:** The highest concentration: 10.4 g/L at 60–70 g/L fructose **Advantages:** Formed a unique ellipse; Low mechanical strength; High hydrodynamic characteristic; High volumetric oxygen transfer	0.22 g/L/h	Chao et al. [194]
**Internal loop airlift with enriched oxygen**	**Advantages:** Unique ellipse was formed; High hydrodynamic characteristic; High volumetric oxygen transfer	0.116 g/L/h	Chao et al. [195]
**Shaking flask with controlled pH/ Hestrin & Schramm medium**	**Attribute:** A membrane-type BC was produced **Advantages:** Varying the net plates number would result in high Young’s modulus and water holding capacity	-	Wu and Li [121]
**Rotating disc bioreactors**			
**A rotating disk bioreactor**	**Attribute:** A consistent product **Advantages:** Produced strong and intact cellulosic matrix, BC pore size of 10–15 μm; High tensile strength	-	Mormino & Bungay [196] Zahan et al. [197]
**Rotating disk bioreactor supported by plastic composites**	**Attribute:** A semi-continuous process **Advantages:** Low mechanical property (Young’s modulus of 372.5 MPa); Low crystallinity: 66.9%; similar thermostability and water content with BC produced by static culture	0.01 g/L/day	Lin et al. [198]
**Rotating disk bioreactor with different additions supported by plastic composites**	**Attribute:** A semi-continuous process **Advantages:** Similar strain but lower stress for carboxymethylcellulose and avicel, respectively; High water retention properties of 98.6–99%; Disc rotation speed and oxygen concentration improved the fermentation process; Fructose concentration was decreased from 50 to 10 g/L; No re-inoculation	0.64 g/slice with 0.8% carboxymethylcellulose and avicel	Lin et al. [199]
**Rotating magnetic field**	**Advantages:** Yield BC with an altered degree of porosity and microstructure; Increased biochemical properties; Positive impact on the growth of bacteria; Increased water retention by 26% as compared to the control sample; high density with tangled and long fibres	-	Fijałkowski et al. [200,201,202,203]
**Other bioreactors for BC production**			
**Spin filter supporting bioreactor**	**Advantages:** BC concentration was increased from 5.65 to 11.52 g/L/140 h; An abundance of Cel + cells were converted into Cel- mutants	0.02 to 0.06 g/L/h	Jung et al. [204]
**Fed-batch principle**	**Advantages:** The gradient of a graph in the load-displacement diagram: (aerosol bioreactor = 34.7 N/10 mm, usual surface culture = 8.9 N/10 mm); High tensile strength: 114 N; High-quality cellulose; the degree of polymerization of BC is 5200; Best time interval: 6 h; BC layer or slices (3–4 cm); Culture box: low cost	-	Hornung et al. [205]
**Biofilm reactor**	**Advantages:** High crystallinity: 93% with a crystal size of 5.2 nm; high biomass density; Water retention ability up to 95 %; better thermal performance	7.05 g/L	Cheng et al. [206]
**Biofilm reactor with additives**	**Advantages:** Continuous BC production; High biomass density; High Young’s modulus and tensile strength; High crystallinity: 80% with a crystal size of 4.2 nm; potential application of BC paper sheets	13 g/L	Cheng et al. [207]
